# Improving graphs of cycles approach to structural similarity of molecules

**DOI:** 10.1371/journal.pone.0226680

**Published:** 2019-12-27

**Authors:** Stefi Nouleho Ilemo, Dominique Barth, Olivier David, Franck Quessette, Marc-Antoine Weisser, Dimitri Watel

**Affiliations:** 1 DAVID, Department of Computer Science, University of Versailles Saint Quentin, Versailles, France; 2 ILV, Department of Chemistry, University of Versailles, Versailles, France; 3 LRI, CentraleSupelec, Paris-Saclay University, Evry, France; 4 ENSIIE, Evry, France; 5 SAMOVAR, Telecom SudParis, Evry, France; Bangladesh University of Engineering and Technology, BANGLADESH

## Abstract

This paper focuses on determining the structural similarity of two molecules, *i.e*., the similarity of the interconnection of all the elementary cycles in the corresponding molecular graphs. In this paper, we propose and analyze an algorithmic approach based on the resolution of the Maximum Common Edge Subgraph (MCES) problem with graphs representing the interaction of cycles molecules. Using the ChEBI database, we compare the effectiveness of this approach in terms of structural similarity and computation time with two calculations of similarity of molecular graphs, one based on the MCES, the other on the use of different fingerprints (Daylight, ECFP4, ECFP6, FCFP4, FCFP6) to measure Tanimoto coefficient. We also analyze the obtained structural similarity results for a selected subset of molecules.

## Introduction

### Motivation

This article focuses on algorithmic approaches to compute the structural similarity of pairs of molecules in large molecular databases. Indeed, in organic chemistry, when a new molecule is designed, it is necessary to determine chemical reactions that can be used to synthesize this target molecule from available compounds. Finding such chemical reactions usually consists in searching in a reaction database (such as REAXYS [1] or ChEBI [2]) for a molecule that is structurally close to the target molecule. As it is sometimes proposed in various existing approaches (see [3] and refs), we assume here that: (i) a molecule is represented by a specific graph encoding it structure, and that (ii) two molecules have a similar structure if they have a similar interconnection of the elementary cycles (typically carbon cycles) of their molecular graphs. The issue is, therefore, to be able to algorithmically select molecules in a reaction database that are structurally similar to a target molecule.

### Background

We consider definitions and notations on graph theory from [4]. Considering a modeling of molecules by graphs [5] or hypergraphs, several definitions and similarity approaches between molecules have already been studied [6], mainly due to the principle stating that structurally similar molecules are expected to display similar properties [7, 8], or to help virtual screening for drug design [9]. Two main approaches are considered to measure the structural similarity of molecules, focusing on specific subgraph problems. The first approach considers the kernel pattern of molecular graphs or hypergraphs, *i.e*., the presence or not of small subgraphs (also called “fingerprints” [10, 11]) belonging to a chosen set of patterns. Such fingerprints are based on cycles or trees, and they are often related to the functional properties of molecules. This approach seems well suited to the classification of molecules according to the properties concerned [12, 13]. It has provided efficient solutions to measure specific molecular similarities, in terms of complexity and performances, but the choice of a significant set of substructures to compare molecules, especially from a structural point of view, is often a difficult problem [3, 6, 14].

The second approach considers the resolution of the problem of finding a Maximum Common Edge Subgraph [6] (MCES) between two graphs. This problem is defined as follows. Considering two graphs *G* = (*V*, *E*) and *G*′ = (*V*′, *E*′), the problem is to find the maximum subgraph of *G* (in terms of number of vertices and edges) being isomorphic to a subgraph of *G*′. This problem is NP-complete [6] and it is initially seen as a generalization of graph isomorphism, with different metrics evaluating the size of this subgraph compared to those of the two graphs to be compared [7, 9, 15, 16]. When consider solving the MCES problem to measure the structural similarity of molecular graphs, two limitations could occur. First, the required computation time is exponential with respect to the number of vertices of the two graphs, which is a major limitation when considering comparing one molecule with all molecules in a database. Second, considering molecular graphs could provide a similarity measure that is not sufficiently focused on structural similarity (*i.e*., the interconnection of elementary cycles of the two molecular graphs).

Note that the last approach consists in measuring distances of weighted editions between two molecular graphs, an edition being an operation of adding or deleting a vertex or an edge in the graph, or changing the label of a vertex. These approaches are notoriously used in the field of bioinformatics [17, 18].

### Contribution

Taking into account the advantages and disadvantages of the two main approaches given above about evaluating the structural similarity of molecules, we investigate here a new approach consisting in computing an MCES on graphs representing the interconnection of cycles in each molecule, *i.e*., with fewer vertices than their molecular graphs. More specifically, we will evaluate the performances of this approach in terms of efficiency and execution time by comparison with a MCES approach on molecular graphs [6] and different fingerprints approach using Tanimoto coefficient [19] on molecular graphs.

As said above, the structure of a molecule can be seen as the interconnection of the cycles in the maximum 2−connected induced subgraph of the molecular graph. A representation of the structure of a molecule based on its cycles has already been proposed and used to classify and characterize sets of molecules [20, 21] and some open service libraries providing specific cycles in molecular graphs [22], are available but such representations have not yet been considered to evaluate the structural similarity of molecules. To this end, we propose to use a novel graph of cycles definition of a molecule best suited for the efficient computation of structural similarities. In this paper, we consider an extension of the graph of cycles given in [23] modeling not only a relevant subset of cycles in a molecule but also their interconnection, whether they share vertices or not. Such a representation can also be seen as the extension of a reduction of the Markush structure of a molecule into a ring/non-ring reduction scheme leading to express the core structure of a molecule [24], for example, to make classification [25]. Our goal is to confirm that this definition of graph of cycles corresponds sufficiently to the intuitive approach followed by a chemist, that the comparison of the graphs of cycles, based on a specific MCES approach, well corresponds to the targeted notion of structural similarity of molecules and that this approach avoids the questions and limitations of the two other approaches considered above.

The rest of the paper is organized as follows. In Section Methods, we give some preliminary definitions of graph theory and molecular graphs. We also define the graph of cycles of a molecule and propose an algorithm to efficiently obtain it for any given molecule. Then, in Section Results and Discussion, we evaluate the performances of using such graphs of cycles (in terms of time computation and pertinence) to measure the similarity of pairs of molecules and compare it to the similarity with different fingerprints (Daylight, Extended-Connectivity FingerPrint ECFP4, ECFP6, Functional-Class FingerPrint 4 and FCFP6) [26] of Tanimoto coefficient.

## Methods

In this section, we first present some definitions of graph theory that will be used in the rest of the paper. Then we present the classic representation of molecules using molecular graphs. Finally, we introduce the graph of cycles, a new representation based on the interconnection of cycles in molecules.

### Preliminaries about cycles in a graph

As said in the previous section, the structural part of a molecule on which we will focus on is mainly based on induced cycles. Thus, to model this structural part, we need first some preliminaries about cycles in graphs.

We consider a simple and undirected labeled graph *G* = (*V*, *E*) with *n* = |*V*| the number of vertices and *m* = |*E*| the number of edges in *E* = {*e*_1_, *e*_2_, …, *e*_*m*_}.

An elementary cycle *c* can be represented by a vector $c=(e_{1}^{c},e_{2}^{c},...,e_{m}^{c})$ where $e_{i}^{c}=1$ iff the edge *e*_*i*_ belongs to *c* otherwise $e_{i}^{c}=0$.

The ***length of a cycle** c* is the number of edges that belongs to the cycle $|c|=\sum_{i=1}^{m}e_{i}^{c}$.

**Definition 1**. *Let us consider two cycles c*_1_ *and c*_2_ *in a graph G with corresponding of vectors* $c_{1}=(e_{1}^{c_{1}},e_{2}^{c_{1}},...,e_{m}^{c_{1}})$ *and* $c_{2}=(e_{1}^{c_{2}},e_{2}^{c_{2}},...,e_{m}^{c_{2}})$. *The **union of the cycles** c*_1_ *and c*_2_, *denoted by c*_12_ = *c*_1_ ⊕ *c*_2_ *is a set of edges given by* $c_{12}=(e_{1}^{c_{1}}⊕e_{1}^{c_{2}},e_{2}^{c_{1}}⊕e_{2}^{c_{2}},...,e_{m}^{c_{1}}⊕e_{m}^{c_{2}})$ *where* ⊕ *is the XOR boolean operation on the* $e_{i}^{c}$, *assuming* 0 *is false and* 1 *is true*.

Since *c*_1_ and *c*_2_ are elementary cycles, then the union of *c*_1_ and *c*_2_ is a union of edge-disjoint cycles by definition of ⊕. A 2−connected component is a maximal (in terms of inclusion) *k*−connected induced subgraph with *k* ≥ 2.

An **isthmus** is an edge of *G* whose deletion strictly increases its number of connected components. An edge is an isthmus if it is not contained in any cycle of *G*. An *isthmus-free graph* is a graph that does not have any isthmus. If a graph *G* has *p* isthmus then its number of 2−connected components is less or equal to *p*; each connected component of a bridgeless graph is 2−edge-connected. The 2−connected components in a graph are connected in *G* by isthmus-chains (a chain in which each edge is an isthmus).

A **generator** *ζ* of a graph *G* is a set of cycles such that for each cycle *c* of *G* there is a set of cycles *c*_1_, *c*_2_, …, *c*_*k*_ in *ζ* such that *c* = *c*_1_ ⊕ *c*_2_ ⊕ … ⊕ *c*_*k*_. The weight of a generator is the sum of the lengths of its cycles. We denote *ζ*^*i*^ a generator of cycles with a weight less than or equal to *i*. A **cycle basis** of *G* is a minimal generator in terms of inclusion. A generator *ζ* of cycles contains cycles such that each edge which belongs at least to a cycle is represented.

A minimum cycle basis of *G* is a cycle basis with minimum weight.

### Molecular graph

**Fig 1:**
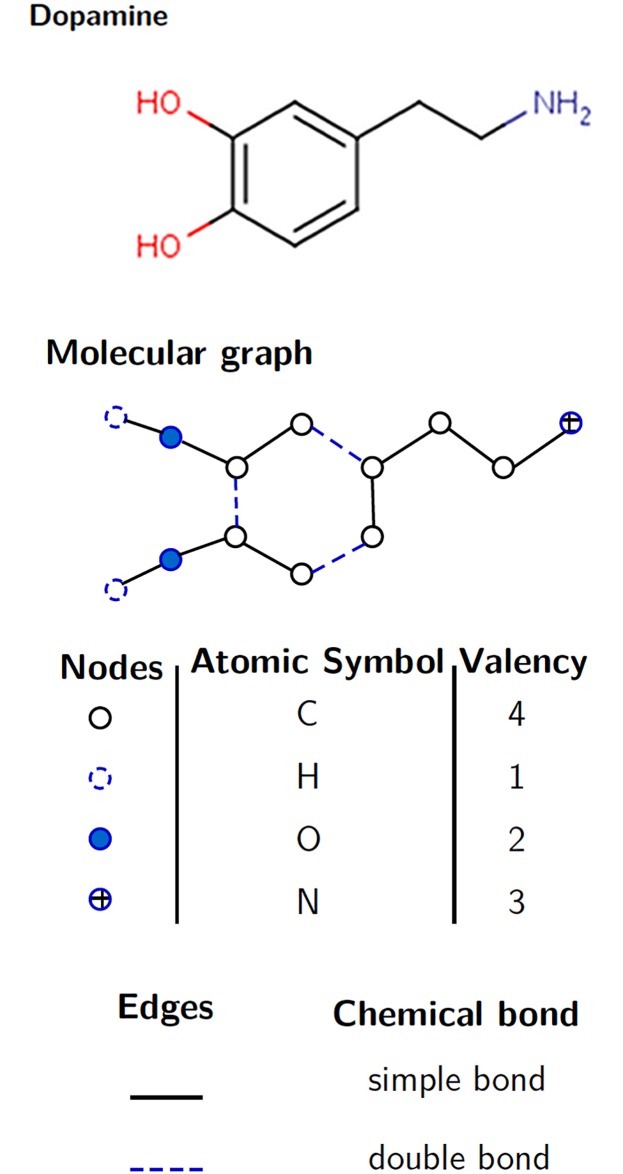
Dopamine and its molecular graph. Different types of nodes according to atomic elements and different types of edges depending on the chemical bond in the molecule.

A molecular graph is an undirected labeled graph *G* = (*V*, *E*) encoding the structural and functional information of the molecule [5]. The set of vertices *V* of *G* encodes atoms and the set of edges *E* encodes the adjacency relationship between atoms in the molecule. Each vertex is labeled by the corresponding chemical element (for example C = Carbon, H = Hydrogen) and each edge is labeled by its type of covalent bond (single −, double =, triple, aromatic). Since hydrogen atoms can be connected at least to one atom, they can be omitted in the representation of a molecule since the valence of each atom is known (see Fig 1). A molecular graph encodes neither the relative spatial arrangement of atoms nor the distance between atoms.

### Representing the cyclic structure of a molecule

The purpose is to model an interconnection between the cyclic parts of the molecule from its molecular graph. We assume that the cyclic part (*i.e*., the *k*-connected components with *k* ≥ 2) describes the structure and the acyclic part describes chemical functions of the molecule, in particular, its possible interactions with other molecules. Thus, the cyclic structure of a molecular graph is based on the interconnection of its induced cycles.

#### Canonical generator

In this subsection, we describe the computation of a canonical generator of cycles for a molecular graph. By canonical, we mean that two isomorphic molecular graphs will produce the same generator.

To get a compact representation of the molecule cycles, we can use minimum cycle bases [27] of the graphs.

**Fig 2:**
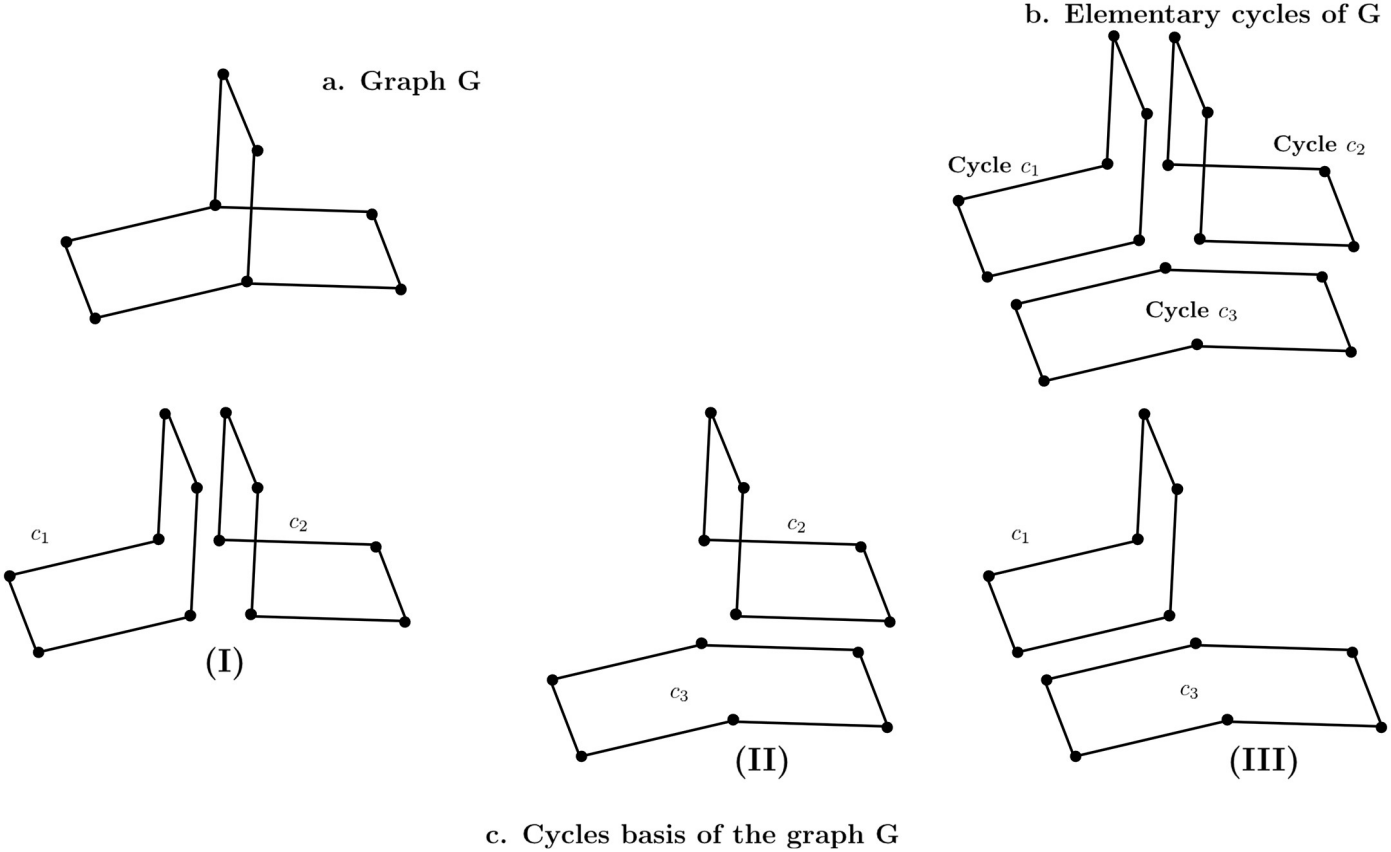
A graph *G*, elementary cycles of *G* and different minimum cycle basis of *G*. A graph can have different minimum cycle basis. From a minimum cycle basis, all elementary cycles of the graph can be generated.

For a graph, we can have more than one minimum cycle basis. It may be difficult to choose a canonical cycle basis to represent the interconnection of cycles because of the non-uniqueness of the cycle basis in a graph (see Fig 2). The goal is to compute similarity on graph of cycles (structural parts). We therefore need the canonical graph of cycles for each molecule, *i.e*., the graph of cycles have to be independent of the vertices labelling and the chosen algorithm to compute a minimum cycle basis. This is the reason why in the definition of graph cycles, cycles are added to a cycle basis to obtain a canonical generator.

The graph of cycles of a given molecule is a graph modeling the interconnection of its elementary cycles. This definition is mainly based on the one proposed in [23].

Vismara [28] reported that the union of minimum cycle basis of the graph is a canonical generator and that the union of minimum cycle basis is the smallest canonical set of cycles that computes the cyclic structure of a graph and the number of cycles of the union of cycle basis can be exponential. The polynomial-time algorithm (complexity of *O*(*ν* × *m*^3^) where *ν* denotes the cyclomatic number) proposed by Vismara computes a compact representation of the potentially exponential-sized set of relevant cycles of a graph. It is said that there is no algorithm to list all the cycles of the union of minimum cycle basis but for molecular graphs, in particular, the compact representation can be replaced by the complete enumeration of the relevant cycles. In the following section, we will introduce an algorithm to compute a canonical generator of a molecular graph.

As we will see in Example 2, for similarity measurement, we will also have to fix an upper bound of the length of the cycles to be considered. Some cycles with a length lower than 8 are sometimes not part of the structural component of the molecule. Thus, we introduce the parameter *j* in the next definition to limit cycles.

Now, we formally define a *canonical generator* for a molecular graph:

**Definition 2**. *Let us consider a generator ζ and an integer j*. *The generator ζ is* ***j*-hierarchical** *if the subset of cycles of ζ with length less than or equal to j can generate all the cycles of lengthless than or equal to j in G*. *A generator ζ is hierarchical iff ζ*_*j*_ *is j*−*hierarchical for every j*.

We denote by *ζ*_*j*_ a *j*-hierarchical set of *ζ*.

**Lemma 1**. *A minimum cycle basis of any graph is hierarchical*.

*Proof*. Let us consider a minimum cycle basis *B*. Assume that *B* is not hierarchical *i.e*. there is an integer *j* such *B*_*j*_ is not *j*-hierarchical.

Since *B*_*j*_ is not *j*−hierarchical, then there is a cycle *c* of length less than or equal to *j* which cannot be generated with *B*_*j*_. Therefore the cycle *c* does not belongs to *B*.

Since *B*_*j*_ is a cycle basis, there is a set of cycles {*c*_1_, *c*_2_, …, *c*_*α*_} in *B* with *c* = *c*_1_ ⊕ *c*_2_ ⊕ … ⊕ *c*_*α*−1_ ⊕ *c*_*α*_. Let us assume that *c*_*α*_ is a cycle of maximum length in the set {*c*_1_, *c*_2_, …, *c*_*α*_}. Since *B*_*j*_ does not generate *c* then the length of *c*_*α*_ is greater than *j*.

The binary operator ⊕ is commutative and associative, so *c*_1_ ⊕ *c*_2_ ⊕ … ⊕ *c*_*α*−1_ ⊕ *c* = *c*_*α*_.

We denote by *B*′ the set of cycles obtain by removing *c*_*α*_ and adding *c* in *B* (*i.e*.*B*′ = *B*\{*c*_*α*_} ∪ {*c*}). As {*c*_1_, *c*_2_, …, *c*_*α*−1_, *c*} ⊂ *B*′, *c*_*α*_ = *c*_1_ ⊕ *c*_2_ ⊕ … ⊕ *c*_*α*−1_ ⊕ *c* and *B* a cycle basis, so is *B*′. The weight of the cycle basis *B*′ is |*B*′| = |*B*| − |*c*_*α*_| + |*c*|. The weight of *B*′ is lower than the weight of *B* (a contradiction because *B* is a minimum cycle basis). Then *B* is hierarchical.

To compute a cycle generator *ζ*_*j*_, we consider an algorithm with less complexity than the one used in [23]: determining the union of all minimum cycles bases. Even if the complexity of such approach can be realistic for molecules of reasonable sizes, it is not convenient to obtain the graph of cycles for all the molecules of a database as Chebi or as Reaxys. Thus, let us consider a molecular graph *G* = (*V*, *E*, *w*_*V*_, *w*_*E*_) that may be disconnected. We define the structural graph of a molecular graph as the maximum subgraph of *G* without any vertex with a degree lower than 2 in the subgraph. We first compute a minimum cycle basis *B* = {*c*_1_, *c*_2_, …, *c*_*k*_} of *G* by executing the Horton algorithm [27] on each 2−connected components of *G*. Then, to obtain a canonical basis, for each pair *c*_*i*_, *c*_*j*_ of cycles in *B*, and for any elementary cycle *c* = *c*_*i*_ ⊕ *c*_*j*_ in *G* and not in *B* such that |*c*| = *max*(|*c*_*i*_|, |*c*_*j*_|), we add *c* in *B*. Finally, *ζ*_*j*_ is the set of all the cycles with a length less than or equal to parameter *j* in *B*.

The complexity of Horton algorithm is polynomial *O*(*n* × *m*^3^) [27] and the complexity of the algorithm that computes a graph of cycles is lower than *O*(*n*^2^ × *m*^3^).

#### Exemple of graph of cycles for a molecular graph

Before defining formally the graph of cycles, we illustrate and explain how to compute a graph of cycles of a molecule on two different examples.

**Fig 3:**
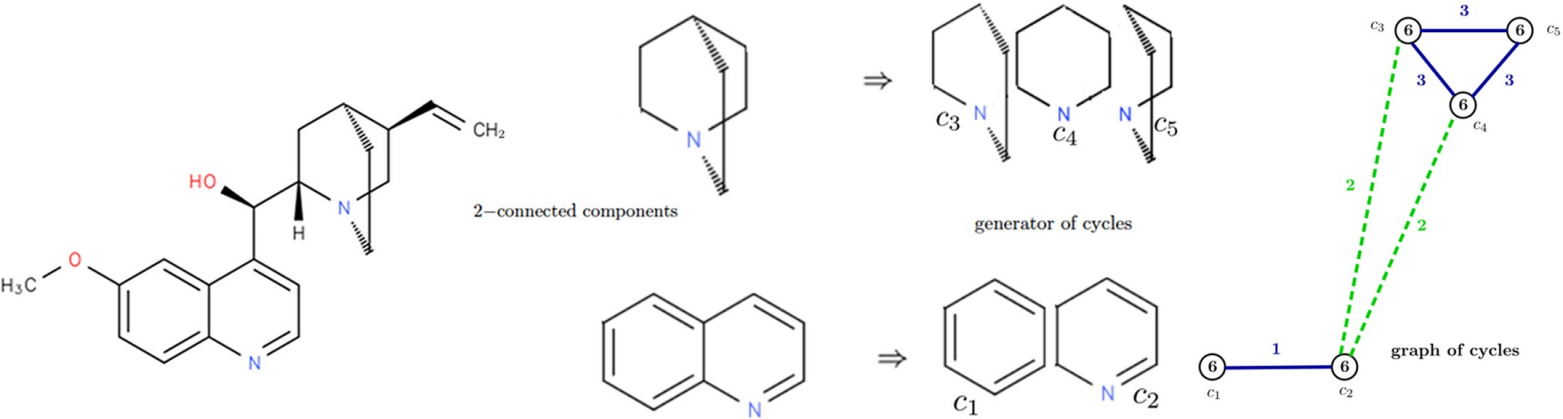
Quinine, 2−connected components, elementary cycles and graph of cycles of quinine. From a graph, we obtain 2−connected components by removing bridges. Then, using a minimum cycle basis, we build a graph of cycle representing the interaction between cycles in a molecular graph.

**Example 1**. Let us consider the molecular graph of quinine, with {*c*_1_, *c*_2_, *c*_3_, *c*_4_, *c*_5_} a canonical generator containing 5 cycles (see Fig 3). These cycles are the vertices of the corresponding graph of cycles. In terms of similarity between molecules, when considering the interaction between cycles in a molecular graph, it is important to distinguish between cycles sharing some vertices (like cycles *c*_1_ and *c*_2_) and cycles linked by a path (like *c*_2_ and *c*_3_). This is why we consider two types of edges in the graph of cycles of a molecule. Firstly, type 1 is used for closed cycles *i.e*. for cycles sharing at least one vertex in the molecular graph. Each edge of type 1 has as label value the number of shared edges. For instance, the plain blue edges in Fig 3 are of type 1. The edge between *c*_1_ and *c*_2_ is equal to 1 because they have one bond in common. Secondly, type 2 is for cycles with a relationship than can be easily broken (the cycles are not closed in the molecular graph). Edges of type 2 have as label value the length of shortest paths between the corresponding cycles in the molecular graph. For example, the dashed green edges in Fig 3 are of type 2. Between *c*_2_ and *c*_3_, the value of the edge of type 2 is 2 (the length of shortest paths between an atom of *c*_2_ and an atom of *c*_3_ in the molecular graph.

The next example illustrates the need of an upper bound of the length of the cycles in the target molecular graph.

**Fig 4:**
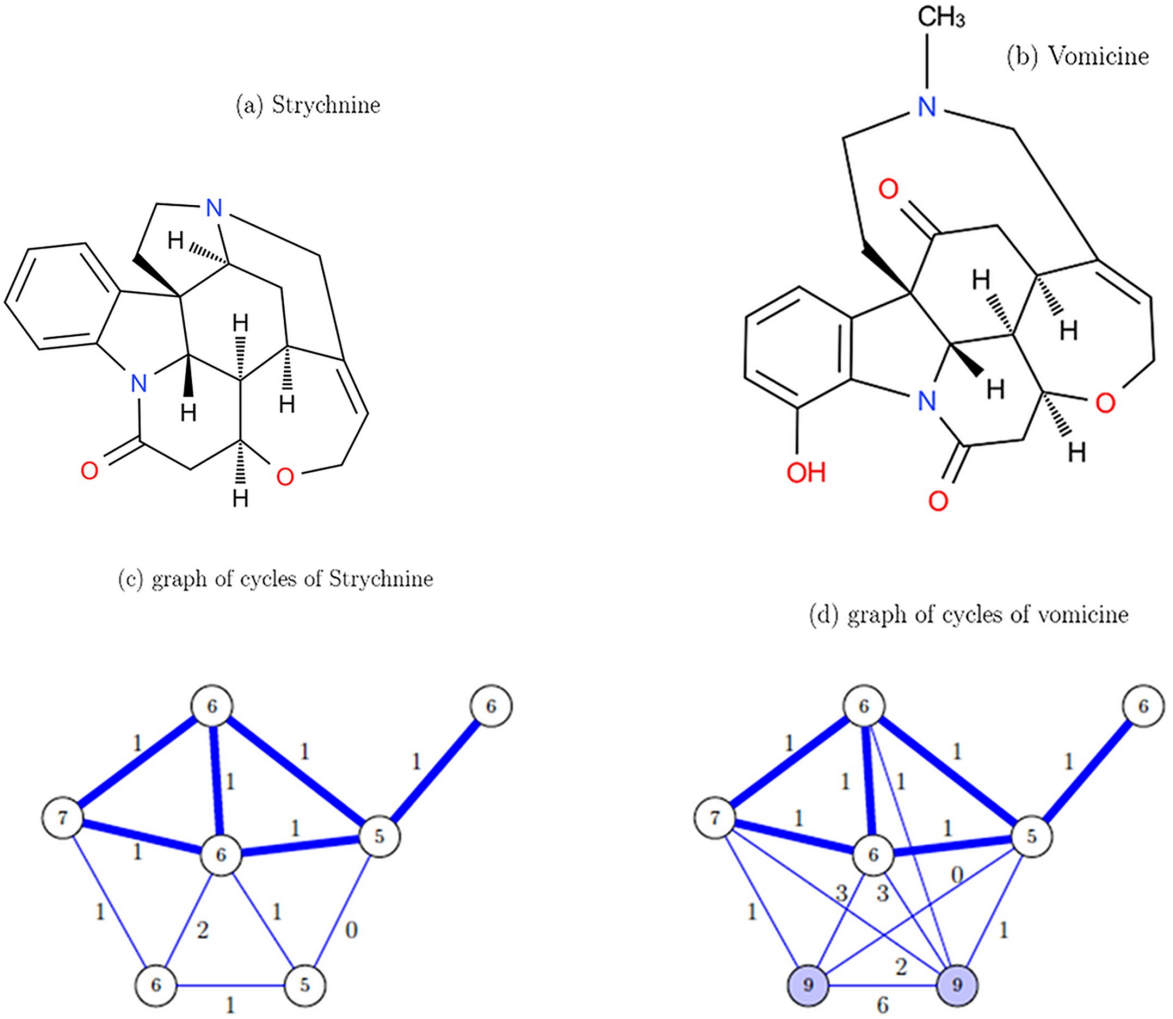
Similar molecules: Strychnine and vomicine with their graph of cycles. Strychnine and Vomicine are structurally similar then their graphs of cycles have to be similar too.

**Example 2**. Let us focus on two molecules considered as structurally similar: strychnine and vomicine. Indeed, as it is illustrated in Fig 4, if we consider all the lengths of cycles in the vomicine, the two molecular graphs do not seem to be similar. But, if we do not consider the cycles of length 9 in the vomicine molecular graph, then the two obtained graphs of cycles are similar. These cycles of length 9 are not cycles mainly involved in the structural of the molecule but they link the structural part with an azote atom. In this case, reducing the graphs of cycles to cycles with a length less than or equal to 7 is relevant, and it will be the case in most situations. It is the reason why we introduce parameter *j* in Definition 2 to limit cycles.

Let us now give the formal definition of a graph of cycles of a molecular graph *G*. Consider a cycle generator *ζ* of *G*. Our goal is to compute a graph *G*^*ζ*^ representing the interconnection between cycles of *G*.

#### Formal definition of graph of cycles

**Definition 3**. *Let G be a molecular graph, an integer j and ζ*_*j*_ *be a j*−*hierarchical generator of cycles in G*. *The **graph of cycles** of G induced by ζ*_*j*_ *is denoted* $G^{\zeta_{j}}=(V^{\zeta_{j}},E^{\zeta_{j}},\mu ,\nu ,\theta )$ *with the edge-set* $E^{\zeta_{j}}=E_{1}^{\zeta_{j}}\cup E_{2}^{\zeta_{j}}$.

- *The vertex-set* $V^{\zeta_{j}}$ *is a j*−*hierarchical generator ζ*_*j*_.
- *The edge-set* $E^{\zeta_{j}}$ *defines the relationship between cycles of* $V^{\zeta_{j}}$ *according to their proximity in G*.
  - $[c_{1},c_{2}]\in E_{1}^{\zeta_{j}}$ *iff c*_1_ *and c*_2_ *belong to the same* 2−*connected components of G and they have at least one common vertex*.
  - $[c_{1},c_{2}]\in E_{2}^{\zeta_{j}}$ *iff c*_1_ *and c*_2_ *belong to different* 2−*connected components and there is a path p from a vertex of c*_1_ *to a vertex of c*_2_ *in G such that all edges of p do not belong to a cycle in* $V^{\zeta_{j}}$.
- *For each vertex* $c\in V^{\zeta_{j}},\mu (c)$ *is the weight of the cycle c*;
- *For each edge* $e\in E_{k}^{\zeta_{j}},$ *ν*(*e*) *is the type of the edge e, ν*(*e*) = *k*;
- *For each edge* $e=[c_{1},c_{2}]\in E^{\zeta_{j}}$, *θ*(*e*) *is the distance from c*_1_ *to c*_2_ *in G*. *If* $e\in E_{1}^{\zeta_{j}}$ *then θ*(*e*) *is the number of common edges between c*_1_ *and c*_2_ *in G*. *If c*_1_ *and c*_2_ *just have one vertex in common then θ*(*e*) = 0. *If* $e\in E_{1}^{\zeta_{j}}$, *then θ*(*e*) *is the length of a shortest path among all the shortest paths between any vertex of c*_1_ *and any vertex of c*_2_ *in G*.

In Example 3, we have *μ*(*c*_1_) = 6 as the length of the cycle *c*_1_, *ν*([*c*_1_, *c*_2_]) = 1, *ν*([*c*_2_, *c*_3_]) = 2 and *θ*([*c*_2_, *c*_3_]) = 2 (the smallest path from a vertex of *c*_2_ to a vertex of *c*_3_ in the molecular graph).

## Results and discussion

In this section, we compare on real cases the performances of three approaches to compute structural similarity of molecules: two approaches using MCES (on molecular graphs (MG) and graph of cycles (GC)), and an approach dealing with molecular graphs based on fingerprints and using the Tanimoto coefficient [19] (TC). We will show that GC can capture the structural similarity of molecules; that MG does not consider cycles when the structural part is considered and that GC and TC do not compute the same kind of similarity even if the results are sometimes similar.

### Similarity using Maximum Commun Edge Subgraph (MCES)

In the MCES approach, we consider the similarity degree defined as follows [6]. Consider two molecular graphs *G*_1_ = (*V*_1_, *E*_1_), *G*_2_ = (*V*_2_, *E*_2_) and a function *π*: *V*_1_ → *V*_2_. A common edge subgraph of *G*_1_ and *G*_2_ denoted *G*_1,2_ = (*V*_1,2_, *E*_1,2_) is a subgraph of *G*_1_ and *G*_2_ that has as many edges as possible and such that if *v* ∈ *V*_1_ and *v*′ ∈ *V*_2_ correspond to the same type of vertex then *v* ∈ *π*(*v*′) (*i.e*., function *π* models the possible correspondences between the vertices of the two graphs). The similarity degree is defined as:

$$\begin{array}{c} \text{sim}\left({\text{G}}_{1},{\text{G}}_{2}\right)=\frac{\left(\right|V_{12}\left|+\right|E_{12}{\left|\right)}^{2}}{\left(\right|V_{1}\left|+\right|E_{1}\left|\right)\times \left(\right|V_{2}\left|+\right|E_{2}\left|\right)} \end{array}$$ (1)

where *G*_1,2_ = (*V*_1,2_, *E*_1,2_) is a maximum commun edge subgraph of *G*_1_ and *G*_2_ maximizing *sim*(*G*_1_, *G*_2_).

In both approaches GC and MG, *π* function is defined as follows.

On one hand, concerning molecular graphs, the function *π* maps atoms of the same type. On the other hand, considering two graphs of cycles $G_{1}^{\zeta_{j}}=(V_{1}^{\zeta_{j}},E_{1}^{\zeta_{j}})$ and $G_{2}^{\zeta_{j^{\prime }}}=(V_{2}^{\zeta_{j^{\prime }}},E_{2}^{\zeta_{j^{\prime }}})$ of two molecules *M*_1_ and *M*_2_, mapping *π* is defined such that for any $v\in V_{1}^{\zeta_{j}},\pi (v)=\{v^{\prime }|v^{\prime }\in V_{2}^{\zeta_{j^{\prime }}}$ *and* ||*v*| − |*v*′|| ≤ 0.2 * *min*(|*v*|, |*v*′|)}. This function *π* allows two cycles, in graphs of cycles, to match if they have a similar length. The value 0.2 has been set experimentally.

When considering graph of cycles, the function *μ* indicates the length of each cycle; the function *ν* indicates the relation between each pair of connected cycles (if they share vertices or not) and the function *θ* gives the label of edges between cycles (see Definition 3).

### Similarity using Tanimoto coefficient

The Tanimoto fingerprint approach [19] has been used as an effective measure of intermolecular similarity. A fingerprint is a structure fragment or feature found within a structure; this approach considers a list of such predefined patterns. Each existing pattern is represented without considering its number of occurrences. There are several types of molecular fingerprints depending on the method by which the molecular representation is transformed: substructure keys-based (MACCS), path-based (Daylight fingerprint) and circular fingerprints (ECFP4, ECFP6, FCFP4, and FCFP6) [10]. Path-based fingerprints analyze all the fragments of a molecule following a path up to a certain number of bonds. In ECFPs and FCFPs are based on the Morgan algorithm and the environment of each atom up to a fixed diameter is recorded. The Tanimoto coefficient *σ* of two molecules *M*_1_ and *M*_2_ is

$$\begin{array}{c} \sigma =\frac{F_{12}}{F_{1}+F_{2}-F_{12}} \end{array}$$ (2)

where *F*_1_, *F*_2_ and *F*_12_ are, respectively, the numbers of fragments in *M*_1_, *M*_2_, and the number of common fragments to molecules *M*_1_ and *M*_2_. Tanimoto coefficient is based on the assumption that similar molecules have similar patterns. This metric does not take account of the connectivity while MCES calculation does; consequently, the two coefficients are not the same and do not compute exactly the same thing in a molecular graph.

### Data

The target database of molecules considered in the present work is a freely available dictionary of small molecular entities called Chemical Entities of Biological Interest ChEBI [2]. This database contains 90, 130 molecules.

We first uniformly select a set ℳ of 10, 000 molecules among the molecules in ChEBI containing at least three cycles (so that the structural similarity has a meaning). We consider a subset ${ℳ}_{S}$ of 500 molecules in ℳ whose molecules are also chosen uniformly.

We focus on three similarity methods: MCES on molecular graphs (MG), MCES on graphs of cycles (GC), and Tanimoto Coefficient (TC). For Tanimoto coefficient, we used Daylight Fingerprint, ECFP4, ECFP6, FCFP4, and FCFP6. Note that to make sure that the MG and GC methods compute the similarity on the structural part of molecules, we remove all the leaves and isthmus in all molecular graphs for MG before computing similarity. We then calculate the similarity with MG for all the pairs of molecules in ${ℳ}_{S}$ and the similarity with GC and TC for all the pairs of molecules in ℳ. Our first goal is to evaluate and compare the performances of the approaches MG, GC and TC on ${ℳ}_{S}$ from three points of view: the execution time required to calculate the measure of similarity for each pair of molecules, the capacity of each approach to distinguish the subset of pairwise of structurally similar molecules (*i.e*., the ones having similar core structures) and finally the capacity of discriminating real similar, medium similar and not similar pairs of molecules. We also analyze the sets of pairwise structurally similar molecules obtained from the MG method in ℳ.

Finally, we select seven molecules in ${ℳ}_{S}$, pairwise not similar and which have different properties of similarity: some have several homologous molecules in the ChEBI database and others not, some are similar to few non-homologous molecules, others to many. For each molecule, we consider the distributions of computed similarities in each of the three approaches for the 90, 130 molecules in ChEBI, and we compare them to the pairwise similar subsets of molecules, induced by these three methods.

The computation of MG and GC has been done on a cluster Intel(R) Xeon CPU E5-260 v3 @2.40GHz with 64G of RAM. To find a maximum clique in a graph to solve MCES, we use a linear program resolved by SCIP [29] (Solving Constraint Integer Programs). Because of the number of molecules in the database (90130 molecules, knowing that many other databases are larger) and since the similarity calculation between two graphs may have an exponential runtime due to the NP-completeness of the problem MCES (indeed, finding an MCES requires to compute a maximum clique in the product graph of the linegraphs induced by the two considered graphs [6]), we have to set a maximum similarity computation time for each pair of molecules. This time depends on the number of vertices of the considered graphs (MG or GC), which is why the computation time GC is small compared to that of MG since a molecular graph contains more vertices than its graph of cycles; this is especially true when the molecules are similar since in this case, the maximum clique is large. For example, if the maximum time for each similarity is 20 seconds, then the whole computation requires ±20 days on the cluster to compare one molecule to all the other ones in ChEBI. As a consequence of the time limitation, some similarities are not computed for some pairs of molecules in the MG context. The source code is available in the S1 Appendix.

For the Tanimoto Coefficient (TC), we used the RDKit software provided by GitHub and SourceForge (http://www.rdkit.org). The construction of the patterns for fingerprints is made in RDKit using the molecular graph of the selected molecule.

### Computation times

We compare the computation times of the three approaches MG, GC, and TC. We first consider the 124, 750 different pairs of molecules in ${ℳ}_{S}$.

For GC similarity, we do not upper bound the similarity computation time for each pair of molecules. We dissociate the computing time needed to compute the graph of cycles of each molecule (which has to be done only once for each molecule) from the time needed to compute the similarity with MCES. Similarly, we also dissociate for TC the time needed to first compute fingerprints from the one needed for similarity calculation.

Table 1 shows that computing the similarity with MCES on GC is faster than MCES on MG. Less than 4% of pairs of molecules can be computed with MG in 1 second/pair. However, 99.79% and 100% of them are computed in less than 0.1 second/pair respectively with GC and TC. The computation times of the different fingerprints (Daylight, ECPF, FCFP) are equivalent, therefore we present the time for Daylight fingerprint only.

**Table 1:** Computation time (in seconds) of similarity with MCES on MG, GC, and with the Tanimoto coefficient.

Time(sec)	[0.0,0.1[	[0.1,0.2[	[0.2,0.3[	[0.3,0.4[	[0.4,0.5[	[0.5,0.6[	[0.6,0.7[	[0.7,0.8[	[0.8,0.9[	[0.9,1.0[
Method										
**MG**	0	70	178	343	283	673	855	767	1026	506
**TC**	124750	0	0	0	0	0	0	0	0	0
**GC**	124491	192	41	9	8	2	4	0	0	0
	[**1,2**[	[**2,3**[	[**3,4**[	[**4,5**[	[**5,6**[	[**6,7**[	[**7,8**[	[**8,9**[	[**9,10**[	[**10,11**[
**MG**	9066	7092	5837	5063	4281	3823	3361	3117	2854	2504
**TC**	0	0	0	0	0	0	0	0	0	0
**GC**	2	0	0	0	0	0	0	0	0	0
	[**11,12**[	[**12,13**[	[**13,14**[	[**14,15**[	[**15,16**[	[**16,17**[	[**17,18**[	[**18,19**[	[**19,20**[	>= **20**
**MG**	2357	2101	2031	1910	1730	1538	1503	1390	1303	57188
**TC**	0	0	0	0	0	0	0	0	0	0
**GC**	1	0	0	0	0	0	0	0	0	0

Note that for MG, 45.48% pairs of molecules were not computed because they were exceeding the maximum time allowed (20 seconds). Recall that for GC and TC we do not consider here the pre-processing time to compute graphs of cycles and fingerprints.

It should be noted that we developed GC and MG in C and the software RDKit used for TC is developed in C++. What is important to note here is the low speed of execution of MG compared to GC and TC.

There is no pre-processing time needed on MCES with MG. Table 2 presents the pre-processing time for ℳ. Over 99.98% fingerprints were computed in less than 0.1 seconds each whereas 8.74% of graph of cycles need more than 0.1 seconds.

**Table 2:** Pre-processing time (in seconds) of graphs of cycles for GC and fingerprints for TC.

Time(sec)	[0.0,0.1[	[0.1,0.2[	[0.2,0.3[	[0.3,0.4[	[0.4,0.5[	[0.5,0.6[	[0.6,0.7[	[0.7,0.8[	[0.8,0.9[	[0.9,1.0[
Method										
**TC**	9999	1	0	0	0	0	0	0	0	0
**GC**	9126	436	113	58	29	37	26	15	10	11
	[**1,2**[	[**2,3**[	[**3,4**[	[**4,5**[	[**5,6**[	[**6,7**[	[**7,8**[	[**8,9**[	[**9,10**[	[**10,11**[
**TC**	0	0	0	0	0	0	0	0	0	0
**GC**	64	24	7	14	4	6	5	5	1	0
	[**11,12**[	[**12,13**[	[**13,14**[	[**14,15**[	[**15,16**[	[**16,17**[	[**17,18**[	[**18,19**[	[**19,20**[	>= **20**
**TC**	0	0	0	0	0	0	0	0	0	0
**GC**	0	2	1	3	2	0	0	0	0	0

Note that these pre-processing computations have to be done only once.

### Compared similarities

#### Confusion matrices

We compare the results on structural similarities with MCES on MG, on GC and TC on the 124, 750 pairs of 500 molecules in ${ℳ}_{S}$.

A confusion matrix is a performance measurement used in machine learning classification [30]. We use it to measure whether the three methods give or not the same results. Table 3 gives the confusion matrix of MG and TC. Each row of the matrix represents the number of pairs of molecules in MG with a value of similarity that is distributed in different columns according to their value in TC.

**Table 3:** Confusion matrices of MG and TC.

**TC**	[**.0,.1**[	[**.1,.2**[	[**.2,.3**[	[**.3,.4**[	[**.4,.5**[	[**.5,.6**[	[**.6,.7**[	[**.7,.8**[	[**.8,.9**[	[**.9,1.0**[	= **1.0**
**MG**											
**[.0,.1[**	1678	1731	259	5	0	0	0	0	0	0	0
**[.1,.2[**	2433	6499	2157	63	6	0	0	0	0	0	0
**[.2,.3[**	1399	9638	4642	343	50	7	4	0	0	0	0
**[.3,.4[**	593	8343	7187	1379	274	90	26	5	8	0	0
**[.4,.5[**	190	3932	5091	1834	652	269	97	34	6	3	1
**[.5,.6[**	53	974	1544	1032	486	366	186	91	30	2	0
**[.6,.7[**	10	150	245	236	242	175	161	101	37	6	0
**[.7,.8[**	2	6	15	30	26	50	62	58	39	16	0
**[.8,.9[**	0	1	10	7	8	21	31	35	22	10	1
**[.9,1.0[**	0	0	1	0	0	0	7	5	1	1	0
= **1.0**	0	0	9	0	0	8	5	4	15	23	5

Confusion matrix of similarity on Molecular Graphs (MG) and Tanimoto Coefficient (TC) Daylight on 67, 589 pairs of molecules. All the pairs that are not computed by MG according to the time limit fixed (20 seconds) are removed. The matrices of confusion of TC/GC and MG/GC are presented the Appendix S1 Table.

The confusion matrix between ECFP4 and FCFP4 shows a strong correlation. Furthermore, the matrices of GC with Tanimoto on Daylight fingerprint and circular fingerprints show similar results (in S2 Table). In the rest of the paper, TC is computed with Daylight fingerprint only.

**Fig 5:**
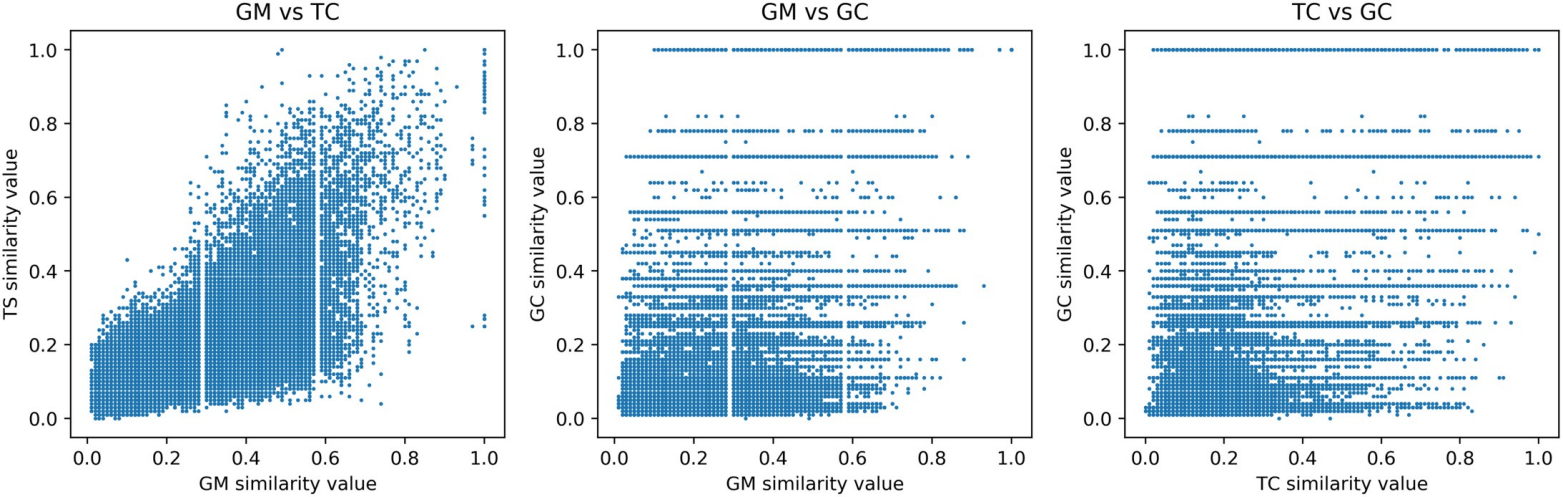
Correlation between (a) MG vs TC, (b) MG vs GC and (c) TC vs GC. We use ${ℳ}_{S}$ and we remove 57, 161 pairs that were not computed after 20 seconds in GC.

We use ${ℳ}_{S}$ to evaluate a correlation between GC, TC, and MG. In Fig 5, the map indicates by a dot each experiment according to the similarity value obtained for each measurement pair. Looking at MG vs TC, we find that dots are aligned around the upper diagonal part. This means that TC and MG provide quite close similar values. There are few dots located in the left upper and right lower part. These dots show pairs of molecules similar on TC but not similar on GC, vice versa.

MG and GC seem to have a lack or no correlation at all (dots are distributed in the space). However, there is a cluster of dots with low similarity values and straight lines of dots for some similarity values on GC. This is explained by the fact that since the graph of cycles here have few vertices, the GC similarity values are closed. The number of possible values for GC is, therefore, smaller than for MG whose graph sizes are larger and for TC for which the calculation is based on a large number of fingerprints sought. We assume that the pairs of molecules belonging to the cluster of dots with low similarity aren’t similar according to all the similarity methods.

We also observe in the middle part of Fig 5 a lack of correlation between MG and GC is transposed on the right part of Fig 5. The GC and TC measurements do not appear to be correlated either.

Moreover, the correlation coefficients between the measurements are respectively equal to 0.1662 between MG and GC and equal to 0.1676 between GC and TC. This value close to zero indicates a low correlation between them. Conversely, the coefficient between MG and TC on Daylight is 0.6028. A coefficient greater than 0.5 indicates a strong correlation.

Evaluating the similarity on ${ℳ}_{S}$ with MG took 15 days for computation. So, the comparison over the 10, 000 molecules of M (near to 50 million pairs of molecules) was done only with TC and GC. However as MG and TC tend to classify pairs of molecules the same way, it is reasonable to assume that comparing MG and GC would return similar results.

**Fig 6:**
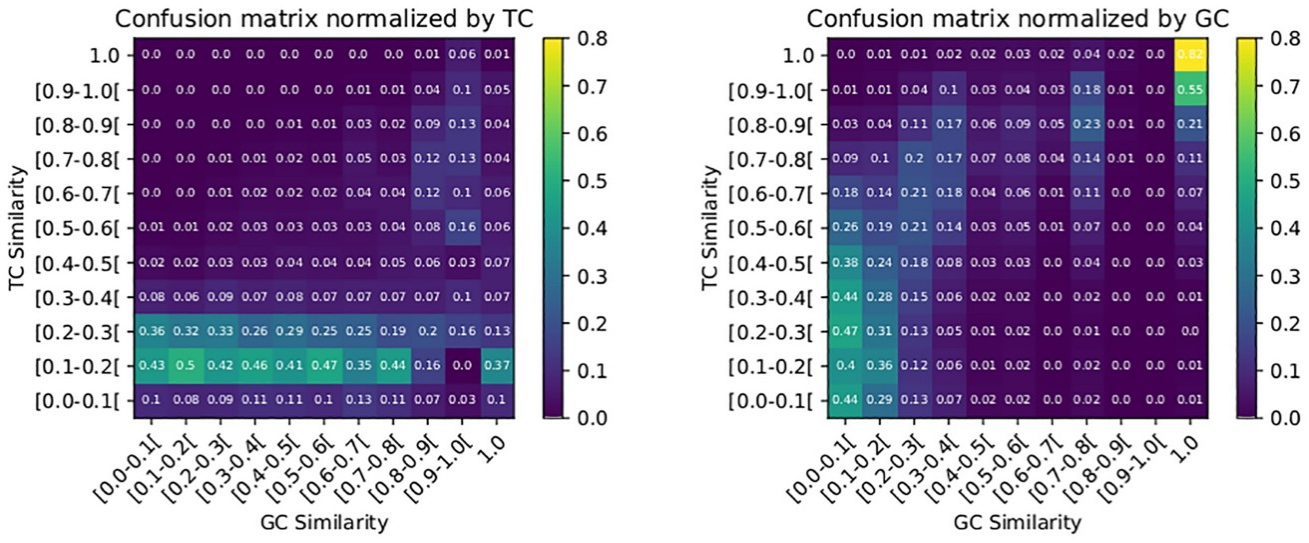
(a)Confusion matrix normalized by TC (b)Confusion matrix normalized by GC. We see it as the conditional probability distribution: given a *x* GC similarity value (resp., TC) and given a randomly selected pair of molecules whose GC similarity is *x* (resp., TC), what is the probability that the similarity TC (or GC) is *y*?

Table 4 is the matrix of confusion of GC and TC on ℳ. The correlation coefficient between GC and TC on ℳ remains low (equal to 0.1559), which indicates that the two measures remain globally uncorrelated. Nevertheless, to have a better view in Fig 6, we normalize the values. In the left (resp. right) part of Fig 6, the normalization is performed with respect to the total number of pairs with a given similarity according to the measurement of GC (resp. TC).

**Table 4:** Confusion matrix of TC Daylight and GC.

**GC**	[**.0,.1**[	[**.1,.2**[	[**.2,.3**[	[**.3,.4**[	[**.4,.5**[	[**.5,.6**[	[**.6,.7**[	[**.7,.8**[	[**.8,.9**[	[**.9,1.0**[	= **1.0**
**TC**											
[**.0,.1**[	2098086	1357516	595708	336076	80188	98966	17215	102408	660	1	45395
[**.1,.2**[	9097068	8085619	2661111	1400400	301127	481123	44955	417304	1524	0	170023
[**.2,.3**[	7646191	5063089	2083353	805793	213752	257548	31931	179596	1886	5	60001
[**.3,.4**[	1635006	1038478	561520	223229	60341	67434	9740	66390	688	3	31877
[**.4,.5**[	467347	298880	221562	98755	31691	41287	4674	48675	555	1	31712
[**.5,.6**[	166688	118032	132009	90406	20376	30049	4311	42683	771	5	28033
[**.6,.7**[	66077	53324	77368	66037	14408	23555	5512	40206	1163	3	27965
[**.7,.8**[	16306	18625	35630	31697	11954	14434	6379	26133	1172	4	19365
[**.8,.9**[	2700	3423	9540	14806	4828	7332	4077	19933	822	4	18191
[**.9,1.0**[	210	570	1620	3962	1162	1677	1099	6899	360	3	21322
= **1.0**	11	30	55	87	107	114	100	191	67	2	3553

Confusion matrix of similarity on TC on Daylight and GC on 49, 995, 000 pairs of ℳ.

In Fig 6(a), we do not detect a correlation between the two metrics. When the conditional probability of TC knowing GC is fixed, the columns are similar. The difference in the two columns [0.8, 0.9[ and [0.9, 1.0[ can be explained by the small number of pairs with GC similarity in the range [0.8;1.0[. This may skew the distributions.

Fig 6(b) shows some links between the two measurements. First, when TC similarity is near to 1.0 then GC similarity is also near to 1.0. On the contrary, when it is weak for TC, it is also weak for GC. As for the intermediate values, there does not seem to be a clear pattern. There are two columns [0.8, 0.9[ and [0.9, 1.0[ containing almost no pairs. This is due to the operation of the GC measurement which tends to rarely give similarity over the interval [0.8;1.0[ (we also see it in Fig 5(b) and 5(c)).

**Fig 7:**
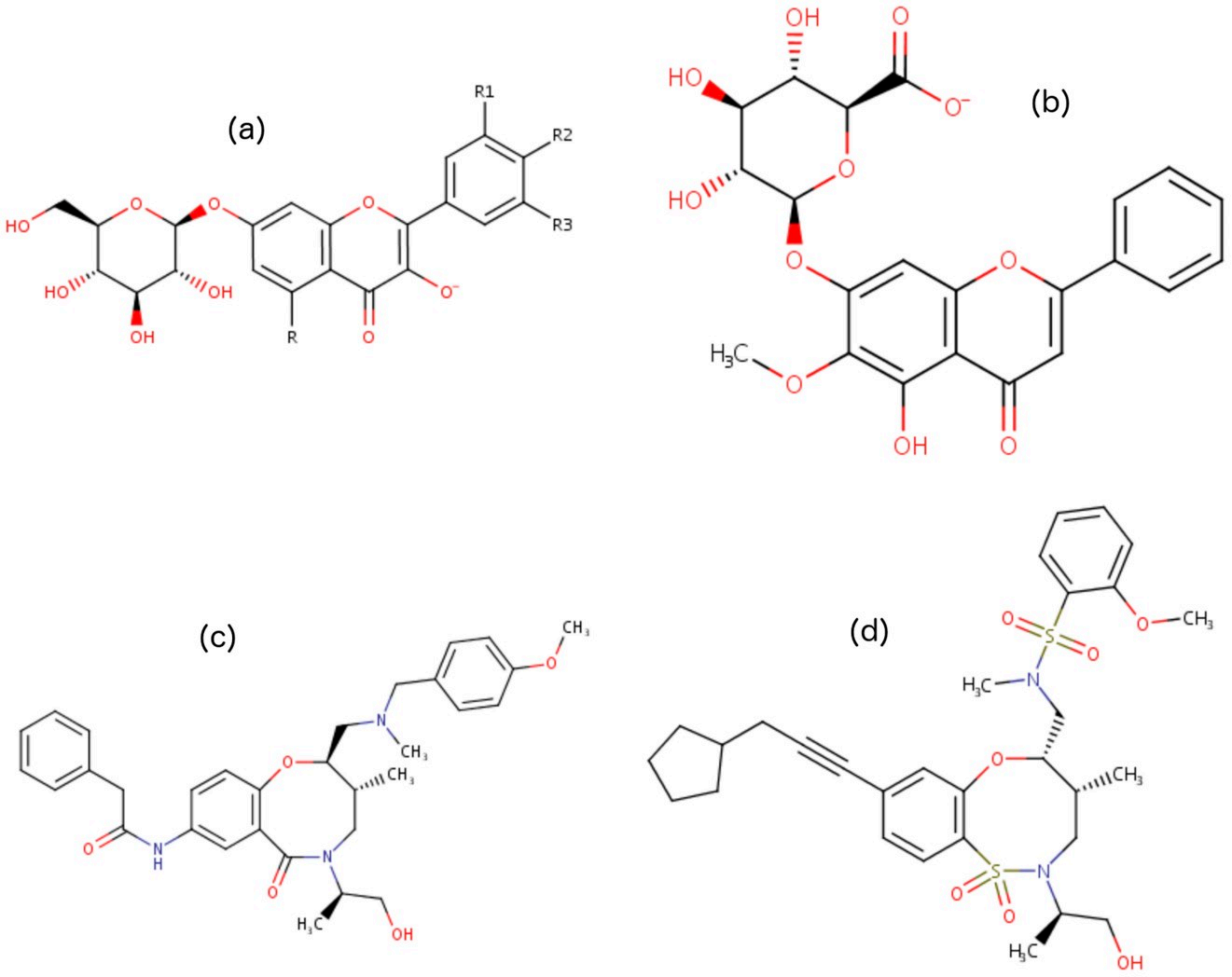
Two molecules of *acid-onion* family and two molecules of *amid* family. Molecule (a) and molecule (b) have a Tanimoto Coefficient equals to 0.25 and a similarity value with graph of cycles equals to 1.0. Molecule (c) and molecule (d) have a Tanimoto Coefficient equals to 0.27 and a similarity value with graph of cycles equals to 1.0. (a)oroxylin A 7-O-*β*-D-glucuronate(b)flavonolate 7-O-*β*-D-glucoside (c) N-[(2R,3R)-5-[(2R)-1-hydroxypropan-2-yl]-2-[[(4-methoxyphenyl)methyl-methylamino]methyl]-3-methyl-6-oxo-3,4-dihydro-2H-1,5-benzoxazocin-8-yl]-2-phenylacetamide (d) N-[[(4R,5S)-8-(3-cyclopentylprop-1-ynyl)-2-[(2R)-1-hydroxypropan-2-yl]-4-methyl-1,1-dioxo-4,5-dihydro-3H-6,1$l{6},2-benzoxathiazocin-5-yl]methyl]-2-methoxy-N-methylbenzenesulfonamide.

All these first results show a strong correlation between the similarity values calculated by the MG and TC methods. On the other hand, the results obtained on ℳ show that there is no correlation between TC and GC, and that this does not depend on the number of fingerprints used by TC, nor on the size of the graphs of cycles. In particular, several pairs of molecules identified as strongly similar by GC (values close to 1) and not by TC corresponds to families of molecules such as *acid-onion* family (with for example molecules (a) and (b) in Fig 7(a) and 7(b)) and the *amid* family (with for example the molecules (c) and (d) in Fig 7(c) and 7(d)), families whose definition implies a structural similarity between their molecules.

#### Connected components

We focus on the subsets of pairwise similar molecules in ${ℳ}_{S}$ induced by the MG, GC, and TC Daylight approaches, for different similarity thresholds. Consider three complete graphs (called in the following similarity graphs) which vertex sets are ${ℳ}_{S}$ and in which each edge is respectively labeled by the similarity computed by MG, TC, and GC of the two connected molecules in the three graphs. Given a similarity threshold *α* ∈ [0, 1], we consider in each similarity graph the connected components induced by the edges with similarity less than or equal to *s*, and their densities (*i.e*. the number of edges over the maximum number edges $\frac{s\cdot (s-1)}{2}$ where *s* is the size of the component).

Table 5 shows that with GC on ${ℳ}_{S}$ we have 24 molecules that have the same structural part with a similarity equal to 1.0. The 8 molecules in MG with threshold of 1.0 are included in the 24 pairs with similarity 1 in GC. For TC, those 24 molecules are in the same connected component only if the threshold is lower than 0.6. In GC, when the threshold is greater than 0.8, the maximum-size connected components do not change and keep a density near to 1.0. We notice that with a threshold lower than 0.4 the average density is high and does not vary more than a gap of 0.1 in GC. However in GC, the density decrease between 0.3 and 0.4 involving that there are many edges with a value in the interval [0.3, 0.4[.

**Table 5:** Connected components, size and average density of connected components on MG, TC, and GC on ${ℳ}_{S}$ depending on threshold.

	MG			TC			GC		
Threshold	#CC	Size Max	Avg Density	#CC	Size Max	Avg Density	#CC	Size Max	Avg Density
**1.0**	461	8	1.0	492	2	1.0	290	24	1.0
**0.9**	455	8	0.98	437	6	0.92	290	24	1.0
**0.8**	387	14	0.84	373	22	0.90	277	24	0.99
**0.7**	265	121	0.78	296	72	0.84	113	339	0.83
**0.6**	150	306	0.85	236	103	0.77	81	383	0.81
**0.5**	59	421	0.62	186	182	0.90	51	435	0.82
**0.4**	21	480	0.16	123	340	0.90	37	450	0.87
**0.3**	15	486	0.30	24	472	0.70	13	488	0.13
**0.2**	9	492	0.43	2	499	0.47	2	499	0.26
**0.1**	4	497	0.51	1	500	0.90	1	500	0.58
**0.0**	1	500	0.53	1	500	1.0	1	500	1.0

*#CC* is the number of connected components with at least 2 molecules. For each threshold, *Size Max* is the size of connected components with the maximum size and *Average Density* is the average density of all the connected components with at least 2 molecules. The density of a connected component is the number of edges over the maximum number edges $\frac{s\cdot (s-1)}{2}$ where *s* is the size of the component.

It appears that the GC approach with a threshold value of 0.8 identifies relatively stable subsets of similar molecules, with an order of magnitude growth over connected component densities. GC do not have similar properties to MG and TC approaches. Thus, the GC approach is the one that most clearly distinguishes between subsets of pairwise similar molecules. To evaluate the quality of these subsets, we focus in the next section on a few target molecules.

### Similarity for selected molecules

In order to complement the large-scale empirical evidence concerning the comparison of the efficiency of the three approaches given in the previous section, in particular through connected components of similarity graphs, and to to illustrate how the GC method performs in practice, we have studied the performance of these approaches in a precise and detailed way by focusing on seven selected molecules in ${ℳ}_{S}$. These molecules have different properties in terms of graphs of cycles and similarities: Quinine, Strychnine, Cholesterol, Manzamine A, Docetaxel Anhydrous, Brevetoxin A and Amphotericin B. The Daylight fingerprint is used for Tanimoto Coefficient. We detail the results obtained on these three last molecules because they include all the results and behaviors found for the seven ones.

#### Docetaxel Anhydrous

**Fig 8:**
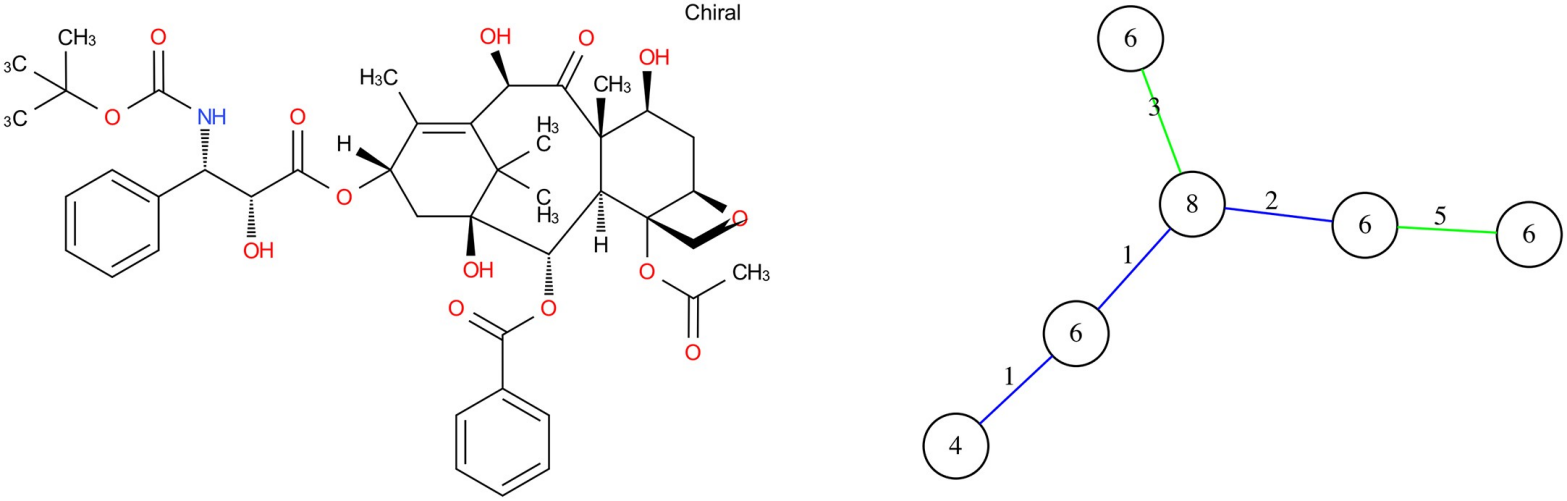
Molecular graph and graph of cycles of Docetaxel Anhydrous. According to the definition of the graph of cycles, we compute the graph of cycles of the molecule Docetaxel anhydrous.

For this molecule, we see that GC gives better results than MG according to similarity calculation and time requested. Docetaxel Anhydrous has a generator of cycles with different lengths (4, 6 and 8). The graph of cycles has 6 vertices and it’s maximum connected subgraph with edges of type 1 is the kernel of this molecule (see Fig 8).

**Fig 9:**
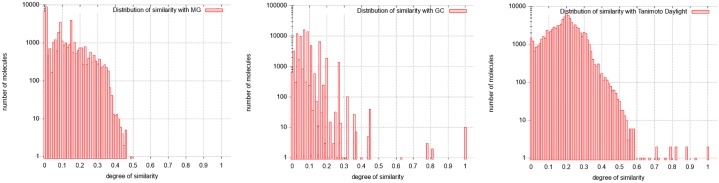
Distribution of similarity on Docetaxel Anhydrous. From left to right: Distribution with molecular graphs (MG), graphs of cycles (GC) and Tanimoto Coefficient (TC).

Fig 9 provides the distributions of similarity on MG, GC, and TC.

**Fig 10:**
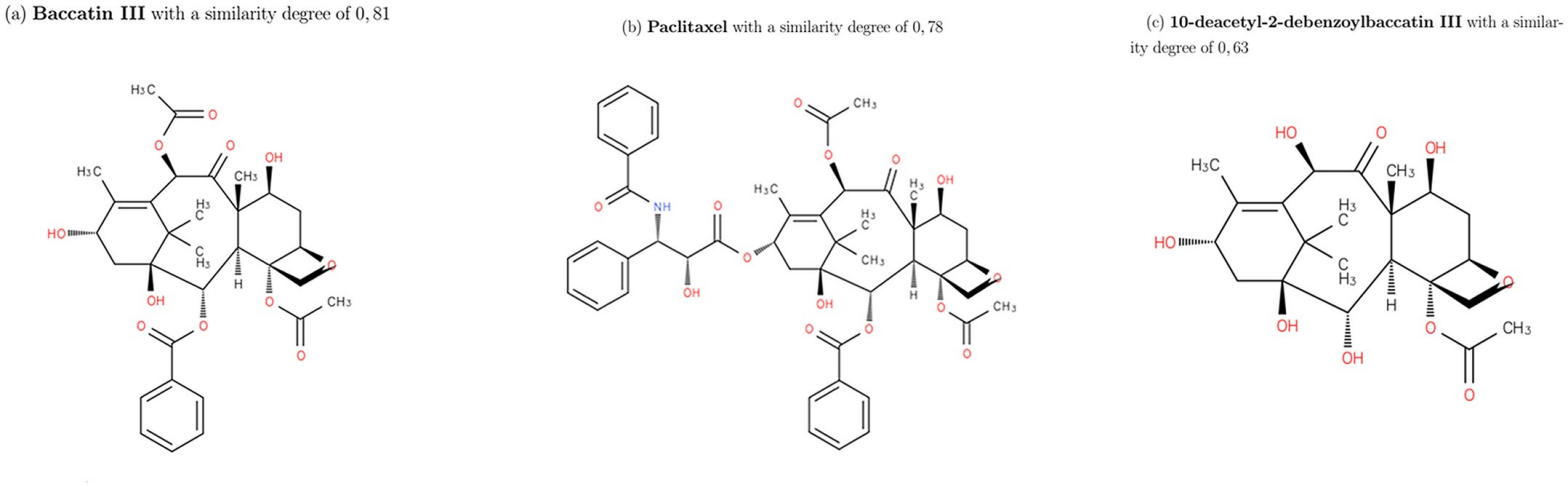
Three molecules similar to docetaxel with GC similarity. From left to right: (a) Baccatin III with a similarity of 0.81, (b) Paclitaxel with a similarity of 0.78 and (c) 10-deacetyl-2-debenzoylbaccatin III with a similarity of 0.63.

According to the distribution of similarity on GC, 4 categories of similar molecules can be extracted:

- 9 molecules are strictly similar to Docetaxel (they are isomers). They have exactly the same graph of cycles (see Fig 10).
- 5 molecules are partially similar; 2 of them have a similarity degree equals to 0.81 differ from Docetaxel only on 1−connected part in MG. Their GC are subgraphs of the graph of cycles of Docetaxel, one cycle linked with an edge of type 2 is missing. The 3 other molecules (with a degree of similarity of 0.78) have the same structure as Docetaxel with more cycles. The GC of these molecules have GC of Docetaxel as subgraph of (they have one cycle more and two edges of type 2).
- 1 molecule is the kernel of Docetaxel. The degree of similarity is 0.63).
- The rest of molecules with a degree lower than 0.45 are not similar to the target molecule.

In the distribution on TC, there are more categories but the first molecules are the same as GC results (GC categories include one or more categories of TC). As similar molecules have the same structure, they also have the same patterns.

In the distribution of similarity on MG, we have set 30 seconds to compute the similarity of two molecules. Over 46, 846 of 90, 130 molecules where not computed (about 51.9%). None of the molecules in top 20 are chemically similar to Docetaxel.

When considering the computing time, TC is the fastest (150 seconds), GC is the second with 1 hour and the last is MG with 10 days.

Finally using the definition related to the connected components given in the previous section, we consider in ℳ the 9 molecules for which the similarity against the Docetaxel Anhydrous molecule is more than 0.7. The largest threshold value for which these molecules appear in a same connected component obtained in ℳ from GC is 0.8; this component contains 11 vertices and its density is 1.0. The GC approach therefore enables to identify a coherent set of molecules being pairwise structurally similar ones associated to Docetaxel Anhydrous. The TC approach identifies in ℳ the same connected component with the same threshold value, but with a much lower density equal to 0.38.

#### Brevetoxine A

**Fig 11:**
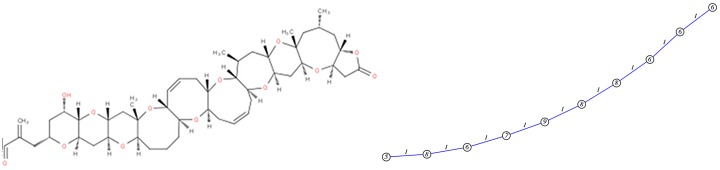
Molecular graph and graph of cycles of Brevetoxine A. According to the definition of the graph of cycles, we compute the graph of cycles of the molecule Brevetoxine A.

The structural part of Brevetoxine A is a chain of cycles. Its particularity is the length of its cycles (5, 6, 7, 8 and 9) with two cycles sharing 0 or 1 common edge in the molecular graph (Fig 11). In this case, we see that GC and TC clearly have not the same ranking. The similarity on GC relies on cycles and TC on patterns.

**Fig 12:**
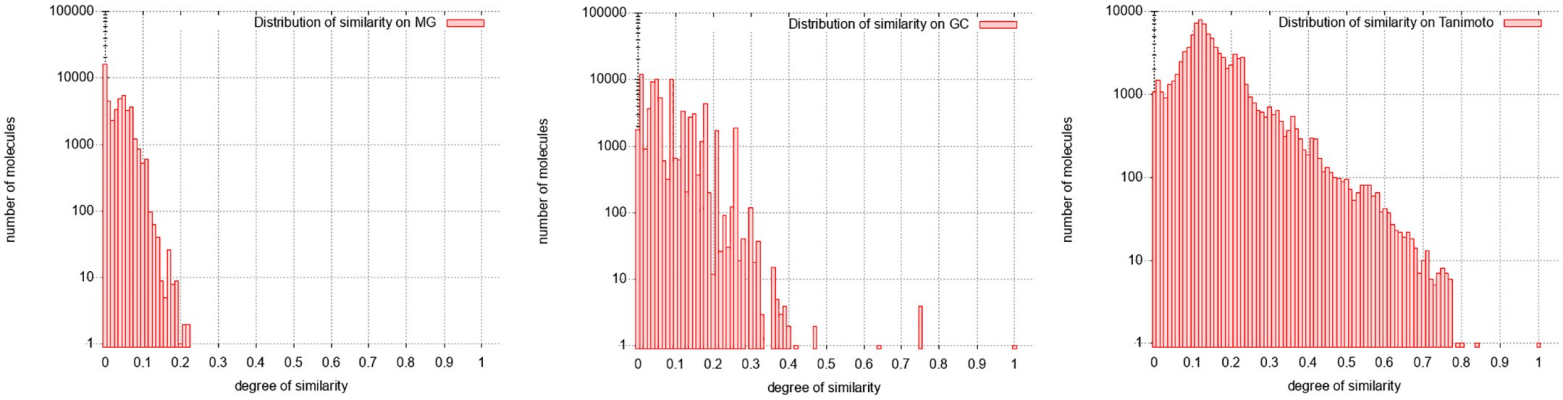
Distribution of similarity on Brevetoxine A. From left to right: Distribution with molecular graphs (MG), graphs of cycles (GC) and Tanimoto Coefficient (TC).

The distribution of similarity of brevetoxin A are shown in Fig 12:

In GC results, we have 3 categories:

- 5 molecules are similar with a degree greater than 0.64. They are members of the same family with Brevetoxin A.
- 2 molecules are similar with a degree equal to 0.47 are partially similar. Their GCs are subgraphs of the GC of Brevetoxin A.
- The rest of molecules with a degree of similarity lower than 0.4 are not similar to Brevetoxin A.

**Fig 13:**
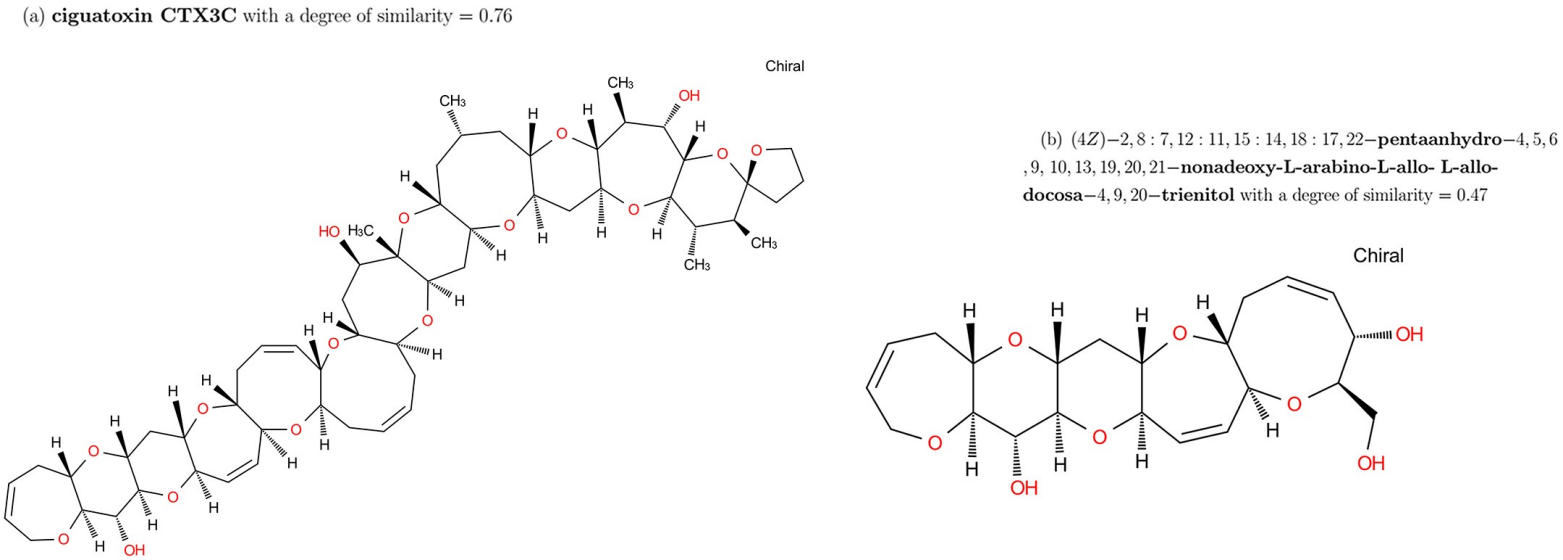
Results of similarity for Brevetoxine A on GC. (a) Ciguatoxin CTX3C with a similarity of 0.76 (b)(4Z)-2,8:7,12:11,15:14,18:17,22-pentaanhydro-4,5,6,9,10,13,19,20,21-nonadeoxy-L-arabino-L-allo-L-allo-docosa-4,9,20-trienitol with a similarity of 0.47. These two molecules share a structural part with Brevetoxin A.

Fig 13 shows two molecules similar according to GC.

**Fig 14:**
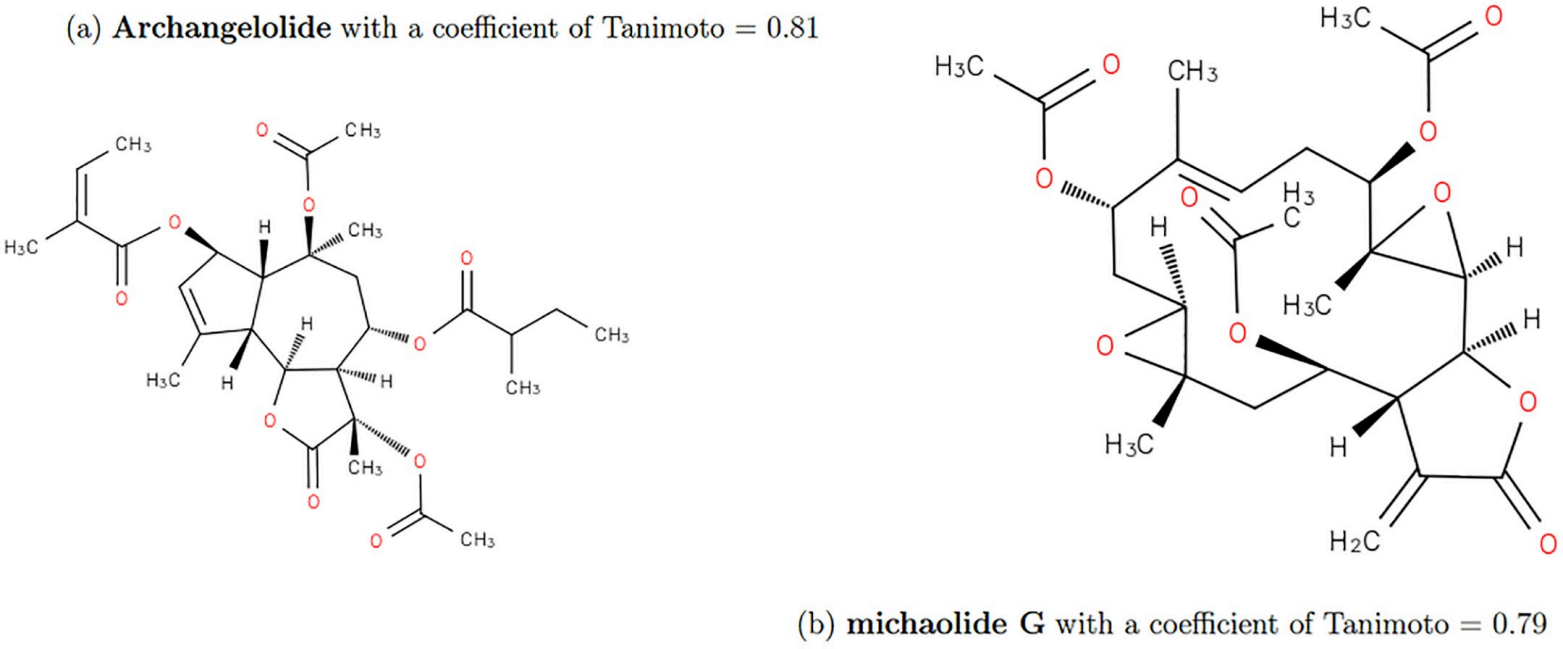
Results of similarity for Brevetoxine A on TC. (a) Archangelolide with a similarity of 0.81 (b) Michaolide G with a similarity of 0.79. These molecules does not have similar structure with Brevetoxin A.

When we look at the TC distribution, the molecule Archangelolide is similar to Brevetoxin A with a Tanimoto coefficient equals to 0.81 (range 2 over 90, 130 molecules). This is because Brevetoxin A does not have many patterns but each pattern occurs several times in the molecule. As a consequence, it affect the results of similarity of this Tanimoto because molecules may be similar without taking account the number of occurences of patterns (appearing 10 times is not the same than one time). This happens with many other molecules (see Fig 14):

For MG, the parameter of time was fixed to 40 seconds. Over 43, 237 of 90, 130 molecules where not computed for MG (47.9%). The first molecule on top 20 is not similar to Brevetoxine A and has a degree of similarity equals to 0.2.

GC similarity gives a better ranking of molecules according to the cycle structure. We observe that the 5 first molecules with a degree lower than 0.5 belong to the same family of Brevetoxin A. Others are less similar to Brevetoxin A according to the cycle structure.

Finally, we consider in ℳ the 3 molecules for which the similarity against the Brevetoxin B molecule is above 0.7. The largest threshold value for which these molecules appear in a same connected component obtained in ℳ from GC is 1; this component contains only these 3 vertices and its density is 1. Approach GC therefore makes it possible to identify a coherent set of molecules being pairwise structurally similar ones associated to Brevetoxine A. The TC approach identifies a connected component in ℳ with 4 vertices, with a maximum threshold equal to 0.92 and with a lower density equal to 0.6.

#### Amphotericin B

**Fig 15:**
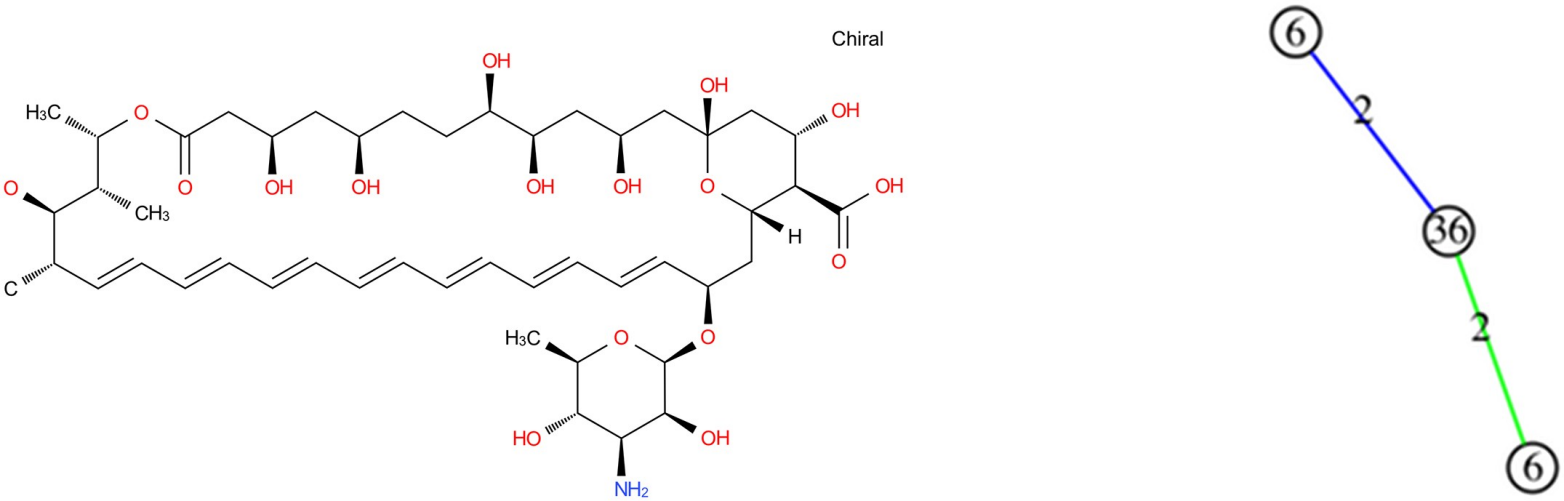
Amphotericin B molecular graph and its graph of cycles. According to the definition of graph of cycles, we compute the graph of cycles of the molecule Amphotericin B.

Amphotericin B has a particular cyclic structure, its minimum cycle basis contains 3 cycles with a particular cycle of length 36 (this cycle belongs to the structural part). The corresponding graph of cycles thus contains 3 vertices (Fig 15).

**Fig 16:**
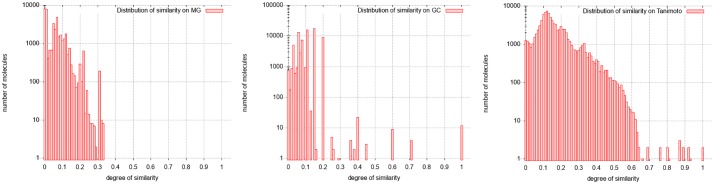
Distribution of similarity on Amphotericin B. From left to right: Distribution with molecular graphs (MG), graphs of cycles (GC) and Tanimoto Coefficient (TC).

The GC distribution of similarities concerns all the molecules of the database. This distribution given in Fig 16 shows 11 molecules fully similar to the target one (degrees of similarity equal to 1), and another distinguished set of molecules being partially similar to it (degrees of similarity equal to 0.6 or to 0.7). The other molecules can be considered as different from the target molecule (similarity lower than 0.5). Thus, the calculation using graphs of cycles discriminates the molecules into three classes, which the molecular graph approach does not do. Moreover, MG approach does not succeed in calculating similarity degrees for several molecules classified as very similar by the GC approach (50, 932 over 90, 130 molecules where not computed; that is 56.5%). This is becaue the required running time is too important; the computation stops because of the set upper bound (20 seconds). Indeed, the required computation time exceeds by far the imposed limit.

**Fig 17:**
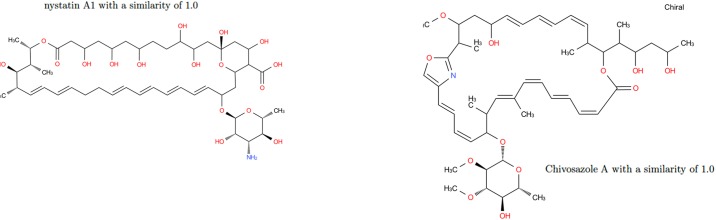
Results of similarity for Amphotericin B on GC. Nystatin A1 (ChEBI id 473992) and Chivosazole A (ChEBI id 80057) with a similarity of 1.0.

Most of the strictly similar molecules provided by the GC approach are either isomers of amphotericin B (amphotericin B methyl ester) or members of the same family (nystatin A1). Amphotericin belongs to the family of antifungal. The other fully similar molecules are not intuitively similar to amphotericin B considering their molecular graphs but the similarity in terms of cycle structure is chemically relevant (Fig 17). The molecules with a similarity degree equal to 0.7 in the GC distribution are the ones for which the graph of cycles has the Amphotericin B grah as subgraph, and the molecules with degree of similarity 0.6 are the ones which graph of cycles is the subgraph of the one of Amphotericin B. Note that these molecules are not discriminated in the MG and TC approach.

**Fig 18:**
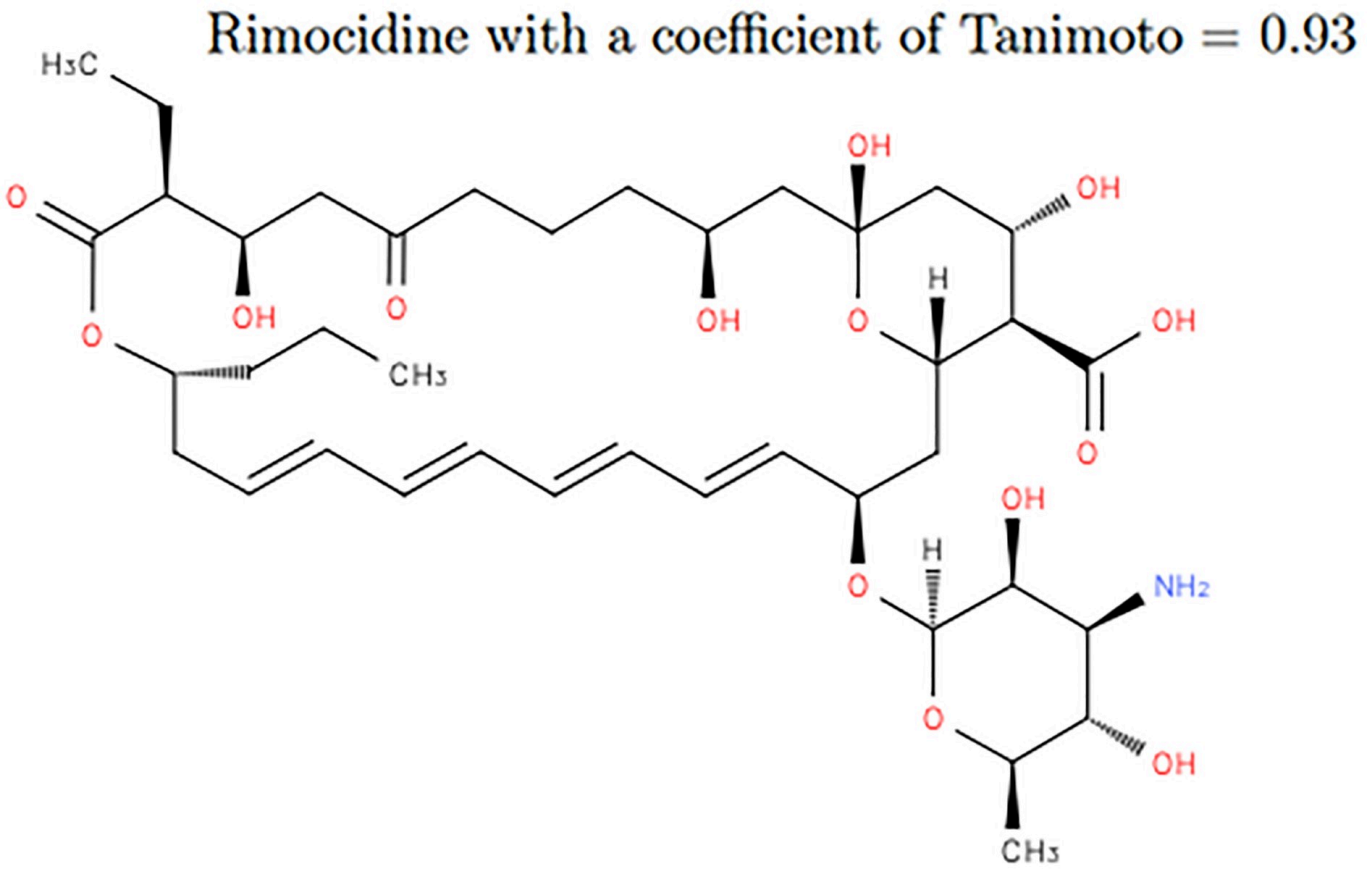
Molecule Rimocidine. Rimocidine (ChEBI id 80106) on TC with a similarity of 0.93.

Molecules similar to Amphotericin B according to TC are also similar in GC except those where the cycle of length 36 is replaced by smaller ones (Fig 18). This is because on Amphoterin B there is the same pattern repeated on this cycle and that Tanimoto does not capture the structure of the molecule. The molecule is actually with a coefficient greater than other molecules having a structure close to the one of Amphotericin B.

Finally, we consider in ℳ the 10 molecules for which the similarity against the Amphotericin B molecule is more than 0.7. The largest threshold value for which these molecules appear in the same connected component obtained in ℳ from GC is 0.7; this component contains 13 vertices and its density is 0.91. The GC approach makes it therefore possible to identify a coherent set of molecules being pairwise structurally similar ones associated to Amphotericin B. The TC approach identifies a connected component in ℳ with 7626 vertices, with a maximum threshold equal to 0.5 and with a lower density equal to 0.04: TC is therefore unable to identify a this coherent set of similar molecules.

The experiments described for this molecule and the two previous ones seem to show that when GC identifies a coherent set of molecules similar to a given molecule, TC also identifies it but in a less precise way, or does not identify them. As indicated at the beginning of the section, the experiments performed for the other selected molecules give similar results.

## Conclusion

Solving MCES problems on graphs of cycles provides a relevant approach for establishing the structural similarity of pairs of molecules. Indeed, the analysis of the performances of the proposed approach shows its efficiency in terms of similarity computation and execution time. As shown by the related component studies in the similarity graphs synthesized in Table 5, which conclusions are not contradicted by the precise study of seven representative cases of molecules, the approach we propose seems very often preferable to discriminate the molecules in terms of structural similarity. Finally, the approach by comparing the graphs of cycles does not require any prior knowledge of structural patterns to be considered in particular to compare the structure of molecules.

An extension of the proposed approach would be to be able to set the length of the cycles (parameter *j*) according to the characteristics of the molecular graph. Indeed, in many cases, taking into account large cycles can distort the similarity measurement because these cycles do not reflect the core structure of the molecules, while in some other cases, taking into account of such cycles is necessary to take all the core structures into account. It seems that the differentiation between these two cases depends, at least in part, on topological properties of the molecular graph, which requires further studies. Finally, the use of other similarity metrics than the resolution of the MCES problem, for example, the use of an editing distance between the graphs of cycles, could also be considered.

## Supporting information

S1 Appendix Source code. (DOCX)

S1 TableMatrices of confusion for similarity MG with GC and TC with MG on ${ℳ}_{S}$.(PDF)

S2 Table Matrices of confusion for similarity MG, GC with TC on ECFP4, ECFP6, FCFP4, and FCFP6. (PDF)
