# Supplementary material for: Improving graphs of cycles approach to structural similarity of molecules
Source: PLoS One. 2019 Dec 27;14(12):e0226680. doi: 10.1371/journal.pone.0226680 (PMC6934298; doi:10.1371/journal.pone.0226680)
Supplement: S1 Appendix — (DOCX) [file pone.0226680.s001.docx]

source code available here :

- MG : <https://github.com/stefinouleho/MCES>
- GC : <https://github.com/stefinouleho/GC_Similarity>
- Tanimoto RDKIT: https://github.com/stefinouleho/Tanimoto_rdkit
- Tanimoto CDK: <https://github.com/stefinouleho/TC_fingerprint>
