# Supplementary material for: Improving graphs of cycles approach to structural similarity of molecules
Source: PLoS One. 2019 Dec 27;14(12):e0226680. doi: 10.1371/journal.pone.0226680 (PMC6934298; doi:10.1371/journal.pone.0226680)
Supplement: S1 Table — (PDF) [file pone.0226680.s002.pdf]

GM \ GC	[.0,.1[	[.1,.2[	[.2,.3[	[.3,.4[	[.4,.5[	[.5,.6[	[.6,.7[	[.7,.8[	[.8,.9[	[.9,1.0[	= 1.0
**[.0,.1[**	**2035**	**911**	**314**	**197**	**64**	**82**	**2**	**68**	**0**	**0**	**0**
**[.1,.2[**	**3647**	**4336**	**1506**	**697**	**127**	**343**	**16**	**344**	**1**	**0**	**141**
**[.2,.3[**	**5537**	**6542**	**1943**	**919**	**204**	**445**	**22**	**337**	**3**	**0**	**131**
**[.3,.4[**	**6429**	**6886**	**2266**	**1178**	**191**	**435**	**25**	**374**	**1**	**0**	**120**
**[.4,.5[**	**4103**	**4562**	**1748**	**919**	**110**	**228**	**4**	**331**	**0**	**0**	**104**
**[.5,.6[**	**1369**	**1579**	**718**	**601**	**42**	**137**	**11**	**237**	**0**	**0**	**70**
**[.6,.7[**	**233**	**336**	**223**	**232**	**33**	**49**	**14**	**138**	**0**	**0**	**105**
**[.7,.8[**	**11**	**30**	**33**	**55**	**11**	**20**	**2**	**73**	**2**	**0**	**67**
**[.8,.9[**	**0**	**7**	**1**	**43**	**0**	**14**	**5**	**9**	**1**	**0**	**66**
**[.9,1.0[**	**0**	**0**	**0**	**1**	**0**	**0**	**0**	**0**	**0**	**0**	**14**
**= 1.0**	**0**	**0**	**0**	**0**	**0**	**0**	**0**	**0**	**0**	**0**	**69**

TABLE 1: Confusion matrix of similarity with Molecular graphs (MG) and Graph Cycles(GC)

TC \ GC	[.0,.1[	[.1,.2[	[.2,.3[	[.3,.4[	[.4,.5[	[.5,.6[	[.6,.7[	[.7,.8[	[.8,.9[	[.9,1.0[	= 1.0
**[.0,.1[**	**5437**	**3165**	**1695**	**1000**	**196**	**280**	**36**	**240**	**0**	**0**	**88**
**[.1,.2[**	**21074**	**19395**	**6815**	**3194**	**696**	**1131**	**68**	**948**	**7**	**0**	**389**
**[.2,.3[**	**20000**	**12962**	**5938**	**1986**	**572**	**649**	**64**	**449**	**5**	**0**	**140**
**[.3,.4[**	**4373**	**2811**	**1598**	**591**	**159**	**172**	**17**	**173**	**0**	**0**	**65**
**[.4,.5[**	**1116**	**788**	**618**	**247**	**68**	**101**	**5**	**112**	**2**	**0**	**67**
**[.5,.6[**	**386**	**286**	**352**	**197**	**49**	**92**	**15**	**115**	**1**	**0**	**55**
**[.6,.7[**	**153**	**85**	**151**	**117**	**47**	**49**	**12**	**126**	**4**	**0**	**61**
**[.7,.8[**	**50**	**45**	**78**	**56**	**36**	**38**	**11**	**63**	**4**	**0**	**45**
**[.8,.9[**	**10**	**13**	**12**	**22**	**11**	**24**	**6**	**55**	**0**	**0**	**34**
**[.9,1.0[**	**1**	**5**	**1**	**4**	**3**	**5**	**2**	**22**	**0**	**0**	**31**
**= 1.0**	**0**	**1**	**0**	**0**	**0**	**1**	**0**	**1**	**0**	**0**	**5**

TABLE 2: Confusion matrix of similarity with Tanimoto Coefficient (TC) and Graph Cycles(GC)
