# Supplementary material for: Improving graphs of cycles approach to structural similarity of molecules
Source: PLoS One. 2019 Dec 27;14(12):e0226680. doi: 10.1371/journal.pone.0226680 (PMC6934298; doi:10.1371/journal.pone.0226680)
Supplement: S2 Table — (PDF) [file pone.0226680.s003.pdf]

**ECFP4** \ **MG**	**[.0,.1[**	**[.1,.2[**	**[.2,.3[**	**[.3,.4[**	**[.4,.5[**	**[.5,.6[**	**[.6,.7[**	**[.7,.8[**	**[.8,.9[**	**[.9,1.0[**	**= 1.0**
**[.0,.1[**	2802	5766	5352	3809	1623	361	61	3	0	0	0
**[.1,.2[**	860	5323	10328	12495	7943	2571	418	24	14	0	1
**[.2,.3[**	10	64	360	1364	2033	1178	404	54	19	0	4
**[.3,.4[**	1	5	35	200	385	400	202	66	21	2	21
**[.4,.5[**	0	0	7	28	101	177	173	84	32	9	6
**[.5,.6[**	0	0	1	5	19	75	87	41	39	2	5
**[.6,.7[**	0	0	0	2	4	2	15	22	12	2	16
**[.7,.8[**	0	0	0	2	1	0	3	9	8	0	8
**[.8,.9[**	0	0	0	0	0	0	0	1	1	0	7
**[.9,1.0[**	0	0	0	0	0	0	0	0	0	0	1
**=1.0**	0	0	0	0	0	0	0	0	0	0	0

Table 1 : Confusion matrix of similarity with TC (ECFP4 fingerprint) and Molecular Graph(MG) on pairs of $\mathcal{M}_S$.

**ECFP6** \ **MG**	**[.0,.1[**	**[.1,.2[**	**[.2,.3[**	**[.3,.4[**	**[.4,.5[**	**[.5,.6[**	**[.6,.7[**	**[.7,.8[**	**[.8,.9[**	**[.9,1.0[**	**= 1.0**
**[.0,.1[**	3100	7492	8418	6589	3098	785	129	7	2	0	0
**[.1,.2[**	571	3655	7566	10750	7980	2938	637	48	25	0	3
**[.2,.3[**	2	11	91	522	880	747	295	85	23	0	18
**[.3,.4[**	0	0	8	36	123	203	190	86	31	9	10
**[.4,.5[**	0	0	0	2	22	79	85	45	38	2	6
**[.5,.6[**	0	0	0	3	5	12	23	17	18	3	13
**[.6,.7[**	0	0	0	3	1	0	3	14	7	1	9
**[.7,.8[**	0	0	0	0	0	0	1	1	2	0	9
**[.8,.9[**	0	0	0	0	0	0	0	1	0	0	1
**[.9,1.0[**	0	0	0	0	0	0	0	0	0	0	0
**=1.0**	0	0	0	0	0	0	0	0	0	0	0

Table 2 : Confusion matrix of similarity with TC (ECFP6 fingerprint) and Molecular Graph(MG) on pairs of $\mathcal{M}_S$.

**FCFP4** \ **MG**	**[.0,.1[**	**[.1,.2[**	**[.2,.3[**	**[.3,.4[**	**[.4,.5[**	**[.5,.6[**	**[.6,.7[**	**[.7,.8[**	**[.8,.9[**	**[.9,1.0[**	**= 1.0**
**[.0,.1[**	2350	3903	2393	821	180	37	4	1	0	0	0
**[.1,.2[**	1218	6741	10930	10462	4979	1172	145	6	2	0	0
**[.2,.3[**	104	492	2579	5649	5091	1970	373	31	10	0	1
**[.3,.4[**	1	22	156	851	1489	1017	372	43	19	0	8
**[.4,.5[**	0	0	22	107	306	419	263	72	26	6	14
**[.5,.6[**	0	0	3	12	55	116	134	83	47	6	7
**[.6,.7[**	0	0	0	0	7	30	56	42	26	3	17
**[.7,.8[**	0	0	0	3	1	3	15	18	4	0	10
**[.8,.9[**	0	0	0	0	1	0	1	8	11	0	8
**[.9,1.0[**	0	0	0	0	0	0	0	0	1	0	4
**=1.0**	0	0	0	0	0	0	0	0	0	0	0

Table 3 : Confusion matrix of similarity with TC (FCFP4 fingerprint) and Molecular Graph(MG) on pairs of $\mathcal{M}_S$.

FCFP6 \ MG	[.0,.1[	[.1,.2[	[.2,.3[	[.3,.4[	[.4,.5[	[.5,.6[	[.6,.7[	[.7,.8[	[.8,.9[	[.9,1.0[	= 1.0
**[.0,.1[**	2952	6610	5639	3012	994	169	21	3	0	0	0
**[.1,.2[**	718	4507	10106	13403	8611	2646	438	28	14	0	1
**[.2,.3[**	3	41	327	1400	2214	1450	480	60	17	0	12
**[.3,.4[**	0	0	11	82	243	372	252	91	31	8	9
**[.4,.5[**	0	0	0	5	37	99	118	70	44	5	10
**[.5,.6[**	0	0	0	0	8	27	39	29	22	1	13
**[.6,.7[**	0	0	0	1	2	1	13	14	11	1	11
**[.7,.8[**	0	0	0	2	0	0	2	8	5	0	9
**[.8,.9[**	0	0	0	0	0	0	0	1	2	0	4
**[.9,1.0[**	0	0	0	0	0	0	0	0	0	0	0
**=1.0**	0	0	0	0	0	0	0	0	0	0	0

TABLE 4 : Confusion matrix of similarity with TC (FCFP6 fingerprint) and Molecular Graph(MG) on pairs of $\mathcal{M}_S$.

ECFP4 \ GC	[.0,.1[	[.1,.2[	[.2,.3[	[.3,.4[	[.4,.5[	[.5,.6[	[.6,.7[	[.7,.8[	[.8,.9[	[.9,1.0[	= 1.0
**[.0,.1[**	5800856	4628055	1704862	928994	217596	320323	37113	299895	1299	0	138118
**[.1,.2[**	13542024	10147849	3756202	1652451	402882	524843	61623	411175	3183	11	138659
**[.2,.3[**	1527461	996966	679728	315518	60214	77205	10429	93267	1077	2	59748
**[.3,.4[**	270700	204672	143791	80611	30329	51381	7307	57566	1293	3	36875
**[.4,.5[**	47461	47362	64773	57263	19631	29847	7177	44710	1179	3	27714
**[.5,.6[**	6554	10790	25480	28822	7111	14346	4292	27248	883	3	19179
**[.6,.7[**	602	1702	4307	6753	1648	4407	1578	11969	450	2	16297
**[.7,.8[**	32	175	313	716	349	998	362	3951	172	3	11840
**[.8,.9[**	0	14	19	112	145	149	87	600	91	2	7474
**[.9,1.0[**	0	0	1	7	27	20	22	30	33	2	1387
**=1.0**	0	1	0	1	2	0	3	7	8	0	146

TABLE 5 : Confusion matrix of similarity with TC (ECFP4 fingerprint) and Graph Cycles(GC) on $49,995,000$ pairs of $\mathcal{M}$.

ECFP6 \ GC	[.0,.1[	[.1,.2[	[.2,.3[	[.3,.4[	[.4,.5[	[.5,.6[	[.6,.7[	[.7,.8[	[.8,.9[	[.9,1.0[	= 1.0
**[.0,.1[**	8769632	7128993	2561280	1338963	319843	471514	53052	424363	1898	2	179778
**[.1,.2[**	11759471	8399090	3385178	1462978	343153	421818	53629	343318	3309	10	133834
**[.2,.3[**	601664	438823	325858	168000	45473	75384	9148	86615	1566	3	52943
**[.3,.4[**	58267	57703	73239	60026	21787	34019	7617	49147	1153	4	32073
**[.4,.5[**	6129	10878	27504	30821	7076	14084	3894	27337	898	1	19311
**[.5,.6[**	511	1830	5720	8542	1984	5018	1944	13132	472	3	15622
**[.6,.7[**	16	249	649	1726	400	1244	521	4296	193	3	10363
**[.7,.8[**	0	18	46	159	159	394	160	1952	107	2	9100
**[.8,.9[**	0	1	2	30	51	37	22	241	51	2	3780
**[.9,1.0[**	0	0	0	2	7	7	5	11	16	1	527
**=1.0**	0	1	0	1	1	0	1	6	5	0	106

TABLE 6 : Confusion matrix of similarity with TC (ECFP6 fingerprint) and Graph Cycles(GC) on $49,995,000$ pairs of $\mathcal{M}$.

FCFP4 \ GC	[.0,.1[	[.1,.2[	[.2,.3[	[.3,.4[	[.4,.5[	[.5,.6[	[.6,.7[	[.7,.8[	[.8,.9[	[.9,1.0[	= 1.0
**[.0,.1[**	3083074	2154297	906405	503981	121495	159136	23401	167068	1006	0	85220
**[.1,.2[**	11843036	9404407	3255400	1512184	377274	547384	54125	418755	2363	8	141859
**[.2,.3[**	5100115	3667341	1577615	682698	149245	173110	25592	157503	1520	4	66591
**[.3,.4[**	971908	634094	439391	215894	44118	62639	8020	77607	1139	1	48729
**[.4,.5[**	170073	139543	137684	88675	26633	43994	6710	56813	1187	1	38446
**[.5,.6[**	24099	30633	47847	46063	14867	23748	6450	40359	933	3	28167
**[.6,.7[**	3166	6126	13116	16662	4839	10072	3951	20092	842	7	22771
**[.7,.8[**	198	1016	1867	4555	1122	2893	1470	9307	462	0	8952
**[.8,.9[**	20	124	139	459	273	459	208	2742	132	3	10618
**[.9,1.0[**	0	4	11	75	65	80	58	157	65	4	5642
**=1.0**	1	1	1	2	3	4	8	15	19	0	442

TABLE 7 : Confusion matrix of similarity with TC (FCFP4 fingerprint) and Graph Cycles(GC) on pairs of $\mathcal{M}$.

FCFP6 \ GC	[.0,.1[	[.1,.2[	[.2,.3[	[.3,.4[	[.4,.5[	[.5,.6[	[.6,.7[	[.7,.8[	[.8,.9[	[.9,1.0[	= 1.0
**[.0,.1[**	6381828	4693727	1792072	949356	232672	326234	41321	306941	1659	1	141781
**[.1,.2[**	13064245	10143704	3744960	1659493	402509	537577	60318	424861	3075	11	149211
**[.2,.3[**	1609826	1064032	667264	321220	62657	87413	10713	98454	1495	1	57801
**[.3,.4[**	124996	111678	123846	81714	25309	41921	7055	56039	1219	1	38013
**[.4,.5[**	13169	20037	40118	41491	11731	19044	5602	34357	856	4	23339
**[.5,.6[**	1548	3802	9859	13745	3964	8300	3304	18452	706	5	21899
**[.6,.7[**	72	541	1234	3706	806	2368	1327	7186	429	2	8703
**[.7,.8[**	5	63	115	462	201	573	299	3478	140	2	6613
**[.8,.9[**	1	1	8	56	77	76	44	616	62	4	8091
**[.9,1.0[**	0	0	0	4	6	12	8	28	22	0	1811
**=1.0**	0	1	0	1	2	1	2	6	5	0	175

TABLE 8 : Confusion matrix of similarity with TC (FCFP6 fingerprint) and Graph Cycles(GC) on pairs of $\mathcal{M}$.

ECFP4 \ FCFP4	[.0,.1[	[.1,.2[	[.2,.3[	[.3,.4[	[.4,.5[	[.5,.6[	[.6,.7[	[.7,.8[	[.8,.9[	[.9,1.0[	= 1.0
**[.0,.1[**	5514185	8016679	540051	6124	66	2	4	0	0	0	0
**[.1,.2[**	1690204	19285890	9061685	591291	11649	176	2	0	0	0	5
**[.2,.3[**	693	253301	1901094	1433023	223012	10275	209	5	1	0	2
**[.3,.4[**	1	919	97607	434201	298431	50673	2535	150	8	2	1
**[.4,.5[**	0	6	893	38173	157873	125927	22939	1259	42	4	4
**[.5,.6[**	0	0	4	727	18517	67304	48215	9417	512	7	5
**[.6,.7[**	0	0	0	1	210	8721	23288	14519	2841	133	2
**[.7,.8[**	0	0	0	0	1	89	4394	5793	7669	952	13
**[.8,.9[**	0	0	0	0	0	2	57	693	4082	3746	113
**[.9,1.0[**	0	0	0	0	0	0	1	6	22	1315	185
**=1.0**	0	0	0	0	0	0	0	0	0	2	166

TABLE 9 : Confusion matrix of similarity with TC (ECFP4 fingerprint) and TC (FCFP4 fingerprint) on pairs of $\mathcal{M}$.
